# Rotational 3D printing of active–passive filaments and lattices with programmable shape morphing

**DOI:** 10.1073/pnas.2537250123

**Published:** 2026-04-22

**Authors:** Mustafa K. Abdelrahman, Jackson K. Wilt, Yeonsu Jung, Rodrigo Telles, Gurminder K. Paink, Natalie M. Larson, Joanna Aizenberg, L. Mahadevan, Jennifer A. Lewis

**Affiliations:** ^a^John A. Paulson School of Engineering and Applied Sciences, Harvard University, Cambridge, MA 02138; ^b^Department of Mechanical Engineering, Stanford University, Stanford, CA 94305; ^c^Department of Chemistry and Chemical Biology, Harvard University, Cambridge, MA 02138; ^d^Department of Physics, Harvard University, Cambridge, MA 02138; ^e^Department of Organismic and Evolutionary Biology, Harvard University, Cambridge, MA 02138; ^f^Wyss Institute for Biologically Inspired Engineering, Harvard University, Cambridge, MA 02138

**Keywords:** liquid crystal elastomers, rotational 3D printing, shape morphing

## Abstract

Natural filaments, such as proteins, plant tendrils, octopus tentacles, and elephant trunks, can transform into arbitrary three-dimensional shapes that carry out vital functions. Their shape-morphing behavior arises from intricate patterning of active and passive regions, which are difficult to replicate in synthetic matter. Here, we introduce a filament-centric strategy for programmable shape morphing in which intrinsic curvature and twist are directly encoded within multimaterial elastomeric filaments during fabrication. By harnessing rotational multimaterial 3D printing, we directly prescribe the filament’s natural curvature–twist field κ(s) through controlled material distribution and helical liquid crystal mesogen alignment. When heated above their nematic-to-isotropic transition temperature (*T*_NI_), the helically aligned liquid crystal elastomer regions contract along their local director field, while passive regions remain essentially unchanged. This approach enables independent control of bending and torsion at every cross-section along the filament centerline: the principal natural curvatures of the filament along two orthogonal axes as well as the local twist. Next, we printed architected lattices composed of unit cells formed by sinusoidal filaments that either reversibly contract, expand, or exhibit out-of-plane deformations. Discrete elastic rod simulations of Janus filaments with different natural curvatures and twist, which are interconnected within the printed lattices, allow accurate prediction of their observed shape-morphing behavior. By integrating active–passive elastomers, additive manufacturing, and computational modeling, we have created shape-morphing matter with complex programmable responses for applications that rely on adaptive, robotic, or deployable architectures.

## Significance

Natural filaments have exceptional control over curvature and twist enabled by directional responses embedded within their internal structure. To emulate this complex behavior, we use rotational multimaterial 3D printing (RM-3DP) to create composite fibers composed of active liquid crystal elastomers (LCEs) and passive elastomers. By controlling the rotation rate on-the-fly during printing, one can fabricate Janus filaments and lattices with spatially programmable composition, alignment, and shape-morphing behavior, including reversible bending, coiling, and twisting when thermally cycled above and below their nematic-to-isotropic transition temperature. A theoretical and computational framework corroborates our experimental findings and paves the way for a quantitative framework to understand and design shape-morphing filaments and lattices.

Nature is replete with active filaments, e.g., proteins (1, 2), plant tendrils (3–5), octopus tentacles (6), and elephant trunks (7–9), all of which exhibit remarkable transformations from straight forms to curved and twisted geometries that fulfill vital biological functions. Proteins, for instance, fold from a random coil into a specific three-dimensional structure to achieve biological activity. Similarly, plant tendrils helically coil to provide structural support, allowing plants to climb toward sunlight, while octopus tentacles and elephant trunks bend and twist to manipulate objects and facilitate communication. These systems illustrate the intimate coupling between filament geometry and function, arising from the deterministic arrangement of their constituents. For instance, the precise sequence of amino acids determines protein folding pathways, stiff lignified cells drive tendril coiling (3, 10, 11), and the patterned activation of muscles combined with passive strain asymmetry enables the versatile movement of the octopus tentacle or the elephant trunk (9, 12). Inspired by these natural examples, synthetic filaments have been developed that exhibit shape-morphing responses for applications in adaptive materials (13–15), soft robotics (16–18), and biomedical devices (19–21).

The shape-morphing behavior of soft filaments can be programmed either extrinsically via differential deformation patterning, intrinsically through the incorporation of stimuli-responsive polymers, or by using a combination thereof. Extrinsically programmed filaments, such as bilayers with varying coefficients of thermal expansion, yield structures that primarily exhibit bending deformations (22–24). More complex deformations, such as twisting and coiling, can be achieved by spatially patterning filaments with inhomogeneous deformations, such as hydrogels with differential swelling ratios (25–28) or elastomers with varying coefficients of thermal expansion (29). Furthermore, by coupling multiple filaments with disparate responses, one can achieve out-of-plane deformations. Indeed, from a mathematical perspective, at every cross-section, there are three translational and three orientational degrees of freedom for the material axes attached to the centerline. The translational degrees correspond to the axial stretch and two transverse shears, while the orientational degrees are associated with two curvatures and a twist. Together, these degrees of freedom span the Euclidean group SE(3) (30, 31). For slender inextensible filaments, the translational degrees of freedom are difficult to actuate (owing to their large stiffnesses), but the orientational degrees of freedom are relatively easy to actuate, resulting in predominantly rotational deformations, i.e., SO(3) (31). From a practical perspective, pneumatic actuators composed of inflatable hollow tubes can exhibit complex 3D deformations even at the filamentary level. These soft robotic filaments can be rapidly created by soft lithography (32, 33) or bubble casting (34), which enable grasping (35), trajectory matching (36), and locomotion (33). However, the reliance on tethered pneumatic systems limits their applicability.

Untethered soft materials that exhibit shape deformation can be achieved using intrinsically programmed, stimuli-responsive materials. For example, the shape memory effect of polymer networks has enabled the development of self-tying filaments; however, shape deformation is typically not reversible, and shape selection is constrained by the need for a mechanical programming step postsynthesis (21). In contrast, liquid crystal elastomers (LCEs) enable reversible, untethered actuation without requiring a mechanical programming step (37). LCEs are stimuli-responsive polymers that undergo reversible shape transformations for at least 10^6^ cycles (38). At the molecular level, LCEs contain rod-like molecules, known as mesogens, that undergo a transition from a nematic (ordered) state to an isotropic (disordered) state upon heating above their nematic-to-isotropic phase transition temperature ($T_{\mathrm{NI}}$), resulting in large, reversible changes in shape (39). The orientation of the nematic director can be programmed through surface alignment (40), magnetic alignment (41–43), or shear-induced alignment (44). While surface and magnetic alignment enable precise programming of structures that bend and twist, they are limited to thin films (45) (~50 µm) or microscopic structures (100 to 500 µm), respectively (41). Direct ink writing (DIW) offers a facile method for printing LCE filaments with programmable, shear-induced alignment for applications ranging from textiles (46–49) to self-sensing artificial muscles (50–52). However, monolithic LCE filaments are typically limited to contractile actuations along the printing (i.e., alignment) direction. To encode more complex deformations, such as coiling, one must introduce additional processing steps during their fabrication (53–55). More broadly, a direct method to prescribe the intrinsic curvature and twist of individual filaments remains largely unexplored.

**Fig. 1.**
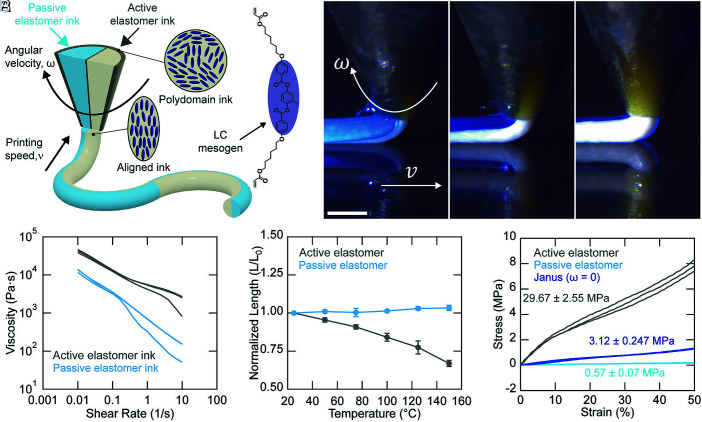
Rotational multimaterial 3D printing of active–passive filaments. (*A*) Schematic of coextrusion of active and passive elastomer inks through a customized 1 mm-diameter nozzle. (*B*) Time-lapse images of ink coextrusion during rotational printing. (Scale bar: 1 mm.) (*C*) Apparent viscosity as a function of shear rate for the active LCE (gray) and passive acrylate (light blue) elastomer inks. (*D*) Normalized length as a function of temperature for pure active LCE (gray) and passive acrylate (light blue) filaments. Data are shown as mean ± SD (n = 3). (*E*) Uniaxial tensile stress–strain curves for an active LCE filament (gray), a passive acrylate filament (light blue), and an active–passive Janus filament printed without rotation (navy blue). All measurements were performed on three independently prepared batches (n = 3).

Here, we harness rotational multimaterial 3D printing (RM-3DP) (56) to fabricate architected elastomer filaments and lattices with programmable shape-morphing behavior. Using customized nozzles with two semicircular channels, active LCE and passive acrylate elastomer inks are coextruded to produce Janus filaments with sharply defined internal interfaces (Fig. 1*A*). Nozzle rotation during printing imposes a helical LCE mesogen orientation and systematically varies the spatial distribution of the two materials along the filament. When heated above *T*_NI_, the active LCE regions contract along the nematic director, while the passive elastomer regions remain largely unchanged, allowing encoding of myriad shape transformations within architected filaments and lattices. Unlike traditional approaches that rely on dense filament networks or thin sheets that morph through differential growth and encode bending through the first and second fundamental forms (25, 28, 29), RM-3DP operates in the filamentary limit and treats each filament as an independent Cosserat rod, allowing direct control over local curvature and twist via prescribed material orientation. Similar to dense and sheet-based systems, RM-3DP can generate smooth, continuous curvature, but it also enables precise control over torsion, multiaxial coupling (i.e., where bending and twisting occur simultaneously), and programmable anisotropy. As simple demonstrations of their potential applications, we fabricated active filters and grippers capable of simultaneously manipulating multiple objects.

## Results and Discussion

Janus filaments are fabricated by coextruding an oligomeric LCE ink, in which rigid mesogens are incorporated along the polymer backbone, and a soft acrylate ink via RM-3DP. A custom nozzle, containing two semicircular channels with a total nozzle diameter of 1 mm, is used during printing (*SI Appendix*, Fig. S1). The liquid crystal mesogens in the active elastomer ink exhibit shear-induced director alignment when printed in the nematic phase (Fig. 1*A*). During printing, the nozzle can be rotated to precisely position each material along the filament length (Fig. 1*B* and Movie S1). The active and passive elastomer inks exhibit shear thinning behavior at 25 °C, the printing temperature (Fig. 1*C* and *SI Appendix*, Fig. S2). Both inks contain acrylate-terminated oligomers ensuring their strong adhesion after UV curing, as confirmed by a 90° peel test. Their peak adhesion strength is 1,289 ± 334 N m^−1^ (*SI Appendix*, Fig. S3). Cohesive failure of the weaker (passive) elastomer indicates that the strength of adhesion between the two elastomers exceeds that of the passive material. The active and passive elastomers exhibit disparate bulk actuation and mechanical properties. When heating the pure controls above $T_{\mathrm{NI}}$, the active LCE filaments undergo a pronounced contraction (57), while the passive filaments remain largely unchanged (Fig. 1*D* and *SI Appendix*, Fig. S4). Normalized lengths of 0.67 ± 0.02 and 1.03 ± 0.02 are observed for the purely active and passive filaments, respectively, upon heating to 150 °C (Fig. 1*E*). The elastic modulus of purely active LCE filaments in the polydomain state is 29.67 ± 2.55 MPa ($T_{g}=-2.71\pm 0.73{}^{\circ}C$), while that of the purely passive filaments is 0.57 ± 0.07 MPa ($T_{g}=-63.12\pm 0.47{}^{\circ}C$) at 25 °C (*SI Appendix*, Figs. S5 and S6). The observed 50-fold difference in elastic moduli between active and passive elastomer provides an additional design parameter for programming filament curvature. Importantly, printed filaments exhibit larger bending responses when the elastic modulus ratio between these features is increased (*SI Appendix*, Fig. S7).

**Fig. 2.**
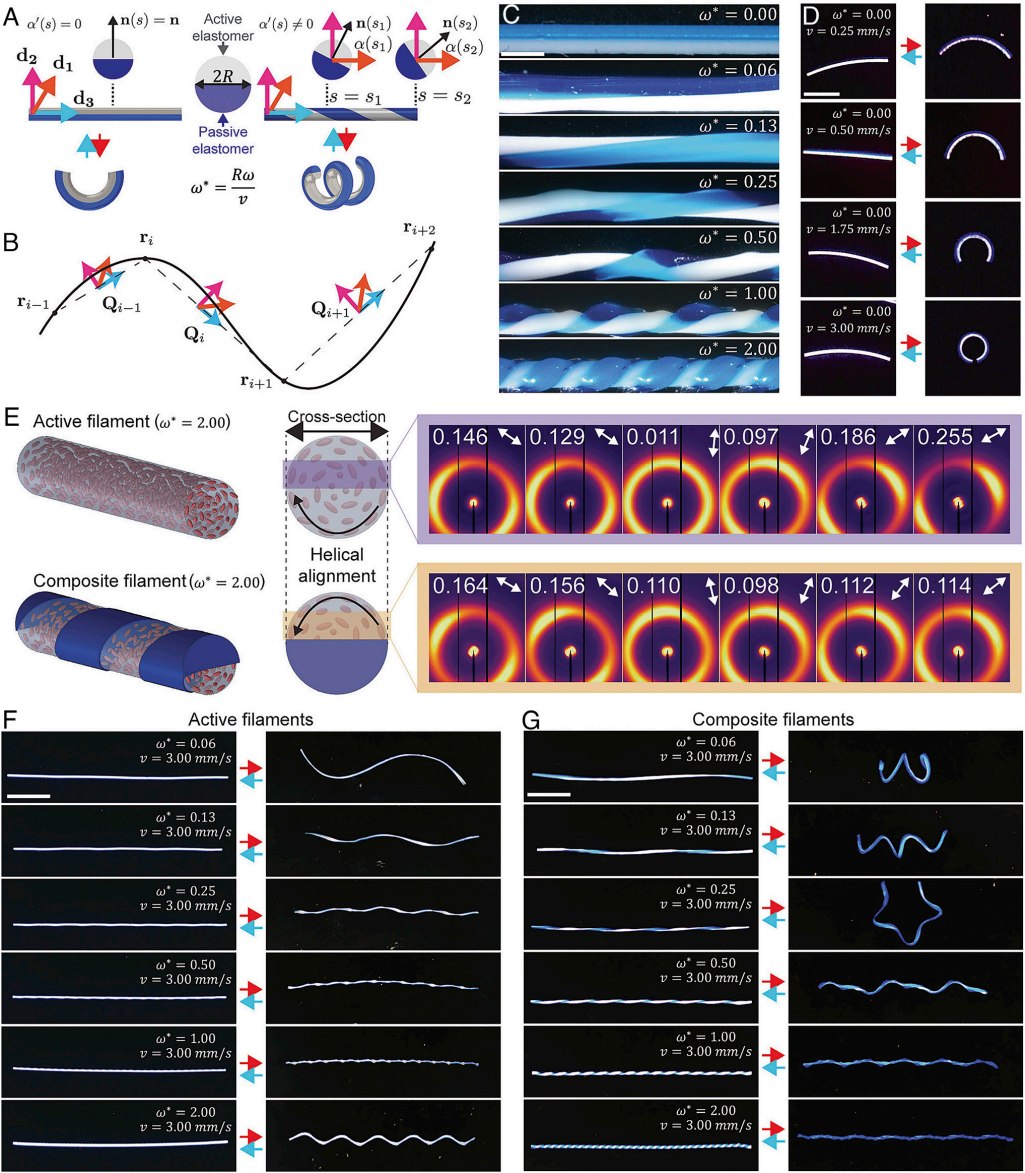
Programmable shape morphing of active–passive filaments. (*A*) Schematic of the material frame and interfacial normal vector n(s). (*B*) Material distribution $\left\{d_{1},d_{2},d_{3}\right\}$ at vertices i-1, i, i+1. (*C*) Optical images of architected filaments printed at varying $\omega^{*}$ with constant ν=3 mm s^−1^. (Scale bar: 1 mm.) (*D*) Filaments printed at $\omega^{*}=0$ with varying print speeds, shown in the initial state and heated states. (Scale bar: 5 mm.) (*E*) Schematic of rotationally printed active LCE and composite filaments, highlighting the helical mesogen orientation imparted by rotational printing. Corresponding 2D WAXS patterns measured across the filament cross-sections reveal the spatial variation of the nematic director orientation. Numbers denote the scalar order parameter (S), and arrows indicate the azimuthal angle of maximum intensity (χmax). Both filaments were printed at $\omega^{*}$ = 2 and ν=3 mm s^−1^. (*F*) Pure active filaments printed at varying $\omega^{*}$ with constant ν=3 mm s^−1^. (Scale bar: 10 mm.) (*G*) Architected composite filaments printed at varying $\omega^{*}$ with constant ν=3 mm s^−1^. (Scale bar: 10 mm.)

We encode shape-morphing behavior by printing active–passive elastomeric filaments with controlled twist and pitch (Fig. 2*A*). In this framework, the primary design variable is not merely material placement, but the spatially varying natural curvature–twist vector κ(s), which defines the intrinsic geometry of the filament independent of external loads. The local material frame $Q\left(s\right)=\left\{d_{1}\left(s\right),d_{2}\left(s\right),d_{3}\left(s\right)\right\}$ defines the filament orientation, with $d_{3}\left(s\right)$ tangent to the centerline and $\left\{d_{1},d_{2}\right\}$ spanning the cross-sectional plane. The interfacial normal vector $n\left(s\right)$ lies within this plane and rotates along the filament according to the prescribed angle α(s). To model this, we represent each filament as a continuous 3D curve, r(s), and introduce a discrete elastic rod (DER) model (Fig. 2*B*) (58, 59). Each discrete filament section consists of a Janus cross-section defined by a normal vector, $n\left(s\right)$, orthogonal to the interface between the active and passive domains in the cross-sectional plane spanned by $\left\{d_{1},d_{2}\right\}$. The orientation of this interface is described by an angle α(s), such that $n\left(s\right)=\cos\left(\alpha \right)d_{1}\left(s\right)+\sin\left(\alpha \right)d_{2}\left(s\right)$, and its spatial rotation dα/ds is directly prescribed through the nozzle rotation rate, ω, through the kinematic relation ω=vdα/ds, where v is the translation speed (Fig. 2 *C* and *D* and Movie S1). A filament with constant α yields a Janus filament, whereas a filament with varying $\alpha \left(s\right)$ introduces a programmed interfacial twist dα/ds along the filament length.

Each filament is modeled as a slender, inextensible, and unshearable rod of length L with a circular cross-section of diameter a. Their deformation can then be described in terms of the relative orientation of each cross-section as a function of location along its axis, and its configuration may be described by the centerline, $r\left(s\right)$, and the material frame $\left\{d_{1}\left(s\right),d_{2}\left(s\right),d_{3}\left(s\right)\right\}$. The elastic energy is then expressed as

$$\begin{array}{c} \epsilon =\frac{1}{2}\int_{0}^{L}{\left(k-\bar{k}\right)}^{T}B(k-\bar{k})ds=\frac{1}{2}\int_{0}^{L}\\ \;\left[B\left({\left(\kappa_{1}-{\bar{\kappa }}_{1}\right)}^{2}+{\left(\kappa_{2}-{\bar{\kappa }}_{2}\right)}^{2}\right)+C{\left(\tau -\bar{\tau }\right)}^{2}\right]ds, \end{array}$$

where $k\left(s\right)=\left\{\kappa_{1}\left(s\right),\kappa_{2}\left(s\right),\tau \left(s\right)\right\}$ is the curvature-twist vector, $\bar{k}\left(s\right)=\left\{{\bar{k}}_{1}\left(s\right),{\bar{k}}_{2}\left(s\right),\bar{\tau }\left(s\right)\right\}$ is the natural curvature-twist induced by differential thermal strains or growth, and is the (diagonal) matrix of stiffnesses B=diag(B,B,C). Here, the bending stiffness $B=EI=E(\frac{\pi a^{4}}{4})$ and the twisting stiffness $C=GJ=G(\frac{\pi a^{4}}{2})$, where E and G are the Young’s modulus and shear modulus, respectively. The equilibrium shape minimizes ϵ; under free boundary conditions, the curvature and twist relax to their natural values, $k\left(s\right)=\bar{k}\left(s\right)$ $\tau \left(s\right)=\bar{\tau }\left(s\right)$, such that bending occurs along the programmed interfacial normal $n\left(s\right)$. We note that our 3D rotational printing platform allows one to control the vector $\bar{k}\left(s\right)=\left\{{\bar{k}}_{1}\left(s\right),{\bar{k}}_{2}\left(s\right),\bar{\tau }\left(s\right)\right\}$ by varying the rate of rotation and the relative ratio of active-to-passive elastomers, providing complete control over the spatial patterning of the orientational degrees of freedom of the filamentary structures.

The dimensionless rotation rate, $\omega^{*}$, quantifies the degree of interfacial twist imposed during RM-3DP, via $\omega^{*}=\frac{Rd\alpha }{\mathrm{ds}}=\frac{Rd\alpha }{\mathrm{dt}}\frac{\mathrm{dt}}{\mathrm{ds}}=\frac{R\omega }{v}$. Filaments printed without rotation ($\omega^{*}=0$) form straight Janus filaments that purely bend upon heating due to differential thermal contraction between the active and passive halves, inducing filament curvature (60). The magnitude of curvature scales with the Weissenberg number, $\text{Wi}=\lambda \dot{\gamma }$, which reflects shear-induced mesogen alignment during extrusion (61). For a fixed nozzle geometry and filament cross-section, increasing the print speed v increases the volumetric flow rate (Q∝v), thereby increasing the shear rate and hence Wi. Accordingly, as v increases from 0.25 to 3.0 mm s^−1^, the order parameter S rises from 0.150 ± 0.002 to 0.230 ± 0.003, leading to stronger contraction and greater curvature (*SI Appendix*, Figs. S8 and S9) (62). Consequently, the maximum curvature of the actuated filament can be tuned from 0.13 ± 0.02 mm^−1^ (for v = 0.25 mm s^−1^) to 0.23 ± 0.01 mm^−1^ (for v = 0.5 mm s^−1^) to 0.47 ± 0.05 mm^−1^ (for v = 1.75 mm s^−1^) to 0.59 ± 0.04 mm^−1^ (for v = 3 mm s^−1^) (Fig. 2*D* and *SI Appendix*, Fig. S10). Notably, under repeated thermal cycling between 25 °C and 175 °C, the filaments exhibit highly reversible curvature with no observable degradation over 100 cycles (*SI Appendix*, Fig. S11). No interfacial debonding, delamination, or slip is observed, even at high curvature, which we attribute to covalent bonds that form between the two acrylate-based (active and passive) elastomer inks.

Programming a spatially varying interfacial twist, α(s), enables control over the twist and out-of-plane bending (or torsion) of the printed filament upon heating above $T_{\mathrm{NI}}$ (Movie S1). Quantitatively, the dimensionless rotation rate, $\omega^{*}$, controls both the pitch and molecular orientation of the LCE filament. As confirmed by two-dimensional (2D) wide-angle X-ray scattering (WAXS) measurements across a filament cross-section, rotational printing imposes a well-defined helical mesogen orientation, whose angle is given by $\varphi \;=\;tan^{-1}\left(\omega *\right)$ (63) (Fig. 2*E* and *SI Appendix*, Fig. S12). Pure LCE (active) filaments inherit this helical director field uniformly across their cross-section and display a characteristic, continuous progression in deformation modes with increasing ω*. At low ω*, shallow helical angles produce bending-dominated morphologies with the largest decrease in end-to-end length ($\begin{array}{c} L/L_{0}\;=\;0.80\;\pm \;0.03\;at\;\omega^{*}\;=\;0.06 \end{array}$) (Fig. 2*F*). As ω* increases, the nematic director rotates, introducing a circumferential contraction component, yielding mixed bending–twisting shapes and a corresponding increase in end-to-end length ($\begin{array}{c} L/L_{0}\;=\;0.86\;\pm \;0.01\;at\;\omega^{*}\;=\;0.25 \end{array}$). Twist reaches its maximum magnitude at ω* = 1, where the director approaches a helical angle of 45° relative to the filament’s centerline and optimally converts uniaxial contraction into torsional strain (64), producing nearly twist-dominated helices with minimal end-to-end length shortening ($\begin{array}{c} L/L_{0}\;=\;0.91\;\pm \;0.01\;at\;\omega^{*}\;=\;1.0 \end{array}$). Further increasing ω* increases the helical angle toward 90°, causing bending to reemerge, leading to end-to-end length shortening ($\begin{array}{c} L/L_{0}\;=\;0.87\;\pm \;0.02\;at\;\omega^{*}\;=\;2 \end{array}$). This “parabolic” variation in normalized length highlights the intrinsic deformation progression of pure LCE filaments upon heating; however, introducing a passive region within the cross-section fundamentally alters this shape-morphing behavior.

Composite filaments inherit the same helical director field but only half of the cross-section is active, resulting in altered shape-morphing behavior. At low ω*, the active region lies predominantly on one side of the filament, establishing a stable cross-sectional strain gradient that strongly favors bending and produces coiled filaments with large end-to-end length shortening ($\begin{array}{c} L/L_{0}\;=\;0.22\;\pm \;0.01\;at\;\omega^{*}\;=\;0.06 \end{array}$) (Fig. 2*G*, *SI Appendix*, Fig. S10, and Movie S2). As ω* increases, the helical angle increases, producing morphologies with coupled bending and twisting, including toroidal coils (ω* = 0.25), reminiscent of DNA supercoiling. However, at high ω*, rapid variation between the active and passive elastomers emerges along the filament’s length, eliminating the geometric condition required for bending. As a result, a stable bending axis cannot form and bending is suppressed. Therefore, twisting becomes the dominant mode of deformation, producing tightly wound helices with nearly no end-to-end length shortening ($\begin{array}{c} L/L_{0}\;=\;0.98\;\pm \;0.02\;at\;\omega^{*}\;=\;2 \end{array}$). This transition from bending-dominated to twisting-dominated morphologies with increasing ω* can be rationalized through the Kirchhoff analogy, which links elastic filament statics to spinning-top dynamics (30, 31), where rapid rotation stabilizes the axis of motion through torsional coupling (65).

The ability to program twisting and coupled bending-twisting deformations could find potential applications as filamentary grippers (16) or synthetic assemblies (13). In each case, the ability to precisely program filament shape is critical for achieving complex, emergent functionality. Furthermore, beyond purely mechanical actuation, emergent functionalities may also be achieved by replacing the passive elastomer with functional materials. For example, incorporating pressure-sensitive adhesive elastomers could enable adaptive gripping, while conductive polymers could impart sensing capabilities. Miniaturization of these filaments could enable further applications, such as colloidal robots (66) or artificial cilia (67). In this work, nozzle sizes are limited due to the resolution limits of our DLP resin printer (~50 µm). Nonetheless, by reducing the nozzle size from 1 mm to 0.5 mm, filament diameters decrease from 600 µm to 300 µm (*SI Appendix*, Fig. S13). However, one caveat is that they must be printed at lower speeds (0.5 mm s^−1^), which reduces the desired shear-induced LCE alignment. We note that although it would be feasible to create nozzles that are 0.1 mm in outer diameter using a higher resolution 3D printer, one must concomitantly reduce the ink viscosity to ensure high printing speed to encode the desired LCE alignment. With this understanding of how printing parameters govern 3D deformation, we next sought to couple rotation with 2D print paths to create spatially patterned, morphable architectures.

**Fig. 3.**
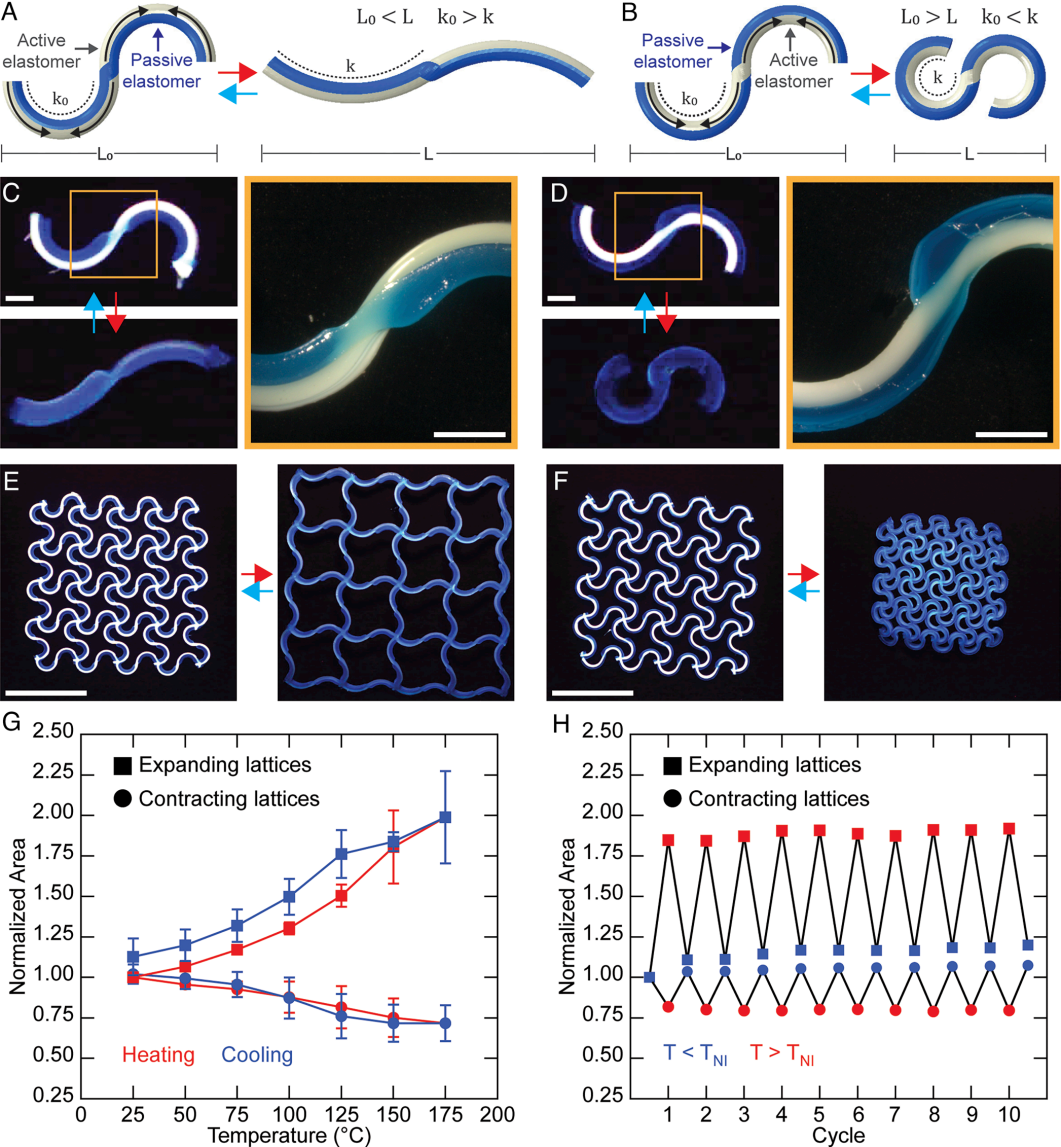
Active–passive lattices with homogeneous shape morphing. (*A*) Schematic of architected filaments with the active elastomer printed on the outer edge, resulting in an increase in filament end-to-end length upon heating. (*B*) Schematic of architected filaments with the passive elastomer printed on the outer edge, resulting in a reduction in filament end-to-end length upon heating. (*C*) Expanding filament below and above $T_{\mathrm{NI}}$. (Scale bar: 1 mm for both images.) (*D*) Contracting filament below and above $T_{\mathrm{NI}}$. (Scale bar: 1 mm for both images.) (*E*) Three-layer lattice composed of expanding filaments, exhibiting global lattice expansion upon heating. (Scale bar: 25 mm.) (*F*) Three-layer lattice composed of contracting filaments, exhibiting global lattice contraction upon heating. (Scale bar: 25 mm.) (*G*) Normalized area as a function of temperature for expanding lattices (squares) and contracting lattices (circles) during heating (red lines) and cooling (blue lines), showing the magnitude of areal changes. Data are shown as mean ± SD (n = 3). (*H*) Normalized area as a function of cycle number for expanding lattices (squares) and contracting lattices (circles) during cyclic heating to 150 °C (red points) and cooling to 25 °C (blue points), demonstrating reversibility.

Because intrinsic curvature is encoded at the filament level, geometric programming propagates hierarchically to the lattice scale, where collective filament interactions give rise to emergent shape transformations. At the filament level, the active LCE elements contract along the direction of director alignment and expand in the transverse direction upon heating above their nematic-to-isotropic transition temperature (*SI Appendix*, Fig. S14). By coupling the aligned LCE elements to passive elements within these Janus filaments, their differential actuation response gives rise to bending. In particular, when the composite filament is printed with an initial curvature and the LCE layer is positioned on the outer (longer) side of the curvature, axial contraction of the LCE reduces the arc-length mismatch between the active and passive layers, leading to filament straightening. As a benchmark, we printed lattices with sinusoidal filaments arranged into homogeneous unit cells, as this geometry enables positioning of the active elastomer on either the outer or inner side of the curvature to drive expansion or contraction (Fig. 3 *A* and *B*). When the active elastomer is placed on the outer curvature, asymmetric contraction upon heating decreases the filament curvature, causing it to straighten and increase its end-to-end distance (Fig. 3*C*). Conversely, positioning the active elastomer on the inner curvature induces an increase in curvature upon heating, shortening the filament’s end-to-end distance (Fig. 3*D*). Upon heating, the normalized end-to-end lengths reach 1.37 ± 0.07 for expanding filaments and 0.64 ± 0.05 for contracting filaments (*SI Appendix*, Fig. S15). Both deformation modes are fully reversible upon cooling.

Extending this concept, we fabricated 4 × 4 lattices composed entirely of either expanding or contracting filaments, yielding lattices that expand or contract uniformly upon heating (Fig. 3 *E* and *F* and Movie S3). Expanding lattices exhibit an increase in area of 99 ± 11%, whereas contracting lattices decrease in area by 28 ± 11% (Fig. 3*G* and Movies S4 and S5). For contracting lattices, deformation is limited by self-contact between neighboring filaments, which leads to a mechanically jammed state that inhibits further contraction and results in a reduced actuation magnitude. We note that adhesion interactions were not observed in these lattices, enabling reversibility over repeated heating and cooling cycles. After ten heating and cooling cycles between 25 °C and 150 °C, contracting lattices return to a normalized area of 1.07 ± 0.03 of their original state, while expanding lattices recover to 1.19 ± 0.05 (Fig. 3*H* and *SI Appendix*, Figs. S16 and S17). Having demonstrated expansion and contraction as independent modes, we next explored spatially combining these responses within a single lattice.

By coupling expanding and contracting regions within the same lattice design, one can drive out-of-plane deformations akin to biological morphogenesis. In nature, the patterning of differential growth or contraction enables the formation of complex 3D structures, such as the folding of organs (68) and the curling of plant tissues (69). Similarly, by architecting lattices composed of unit cells with nontrivial α(s), 3D shape transformations arise from the temperature-driven changes in the natural curvature of sinusoidal filaments. These curvature variations generate configurations that minimize the elastic bending energy ϵ upon heating, producing out-of-plane morphologies with tunable positive or negative Gaussian curvature. Inspired by these principles, we numerically modeled and experimentally validated heterogeneous lattices composed of both expanding and contracting filaments (*SI Appendix*, Fig. S18). We modeled these lattices as networks of DERs, where each sinusoidal filament connects four branch nodes to form a repeating unit cell. At a branch node where multiple sinusoidal edges meet, the total bending and twisting energies are computed by summing the contributions from all unique pairs of connecting edge segments (70).

**Fig. 4.**
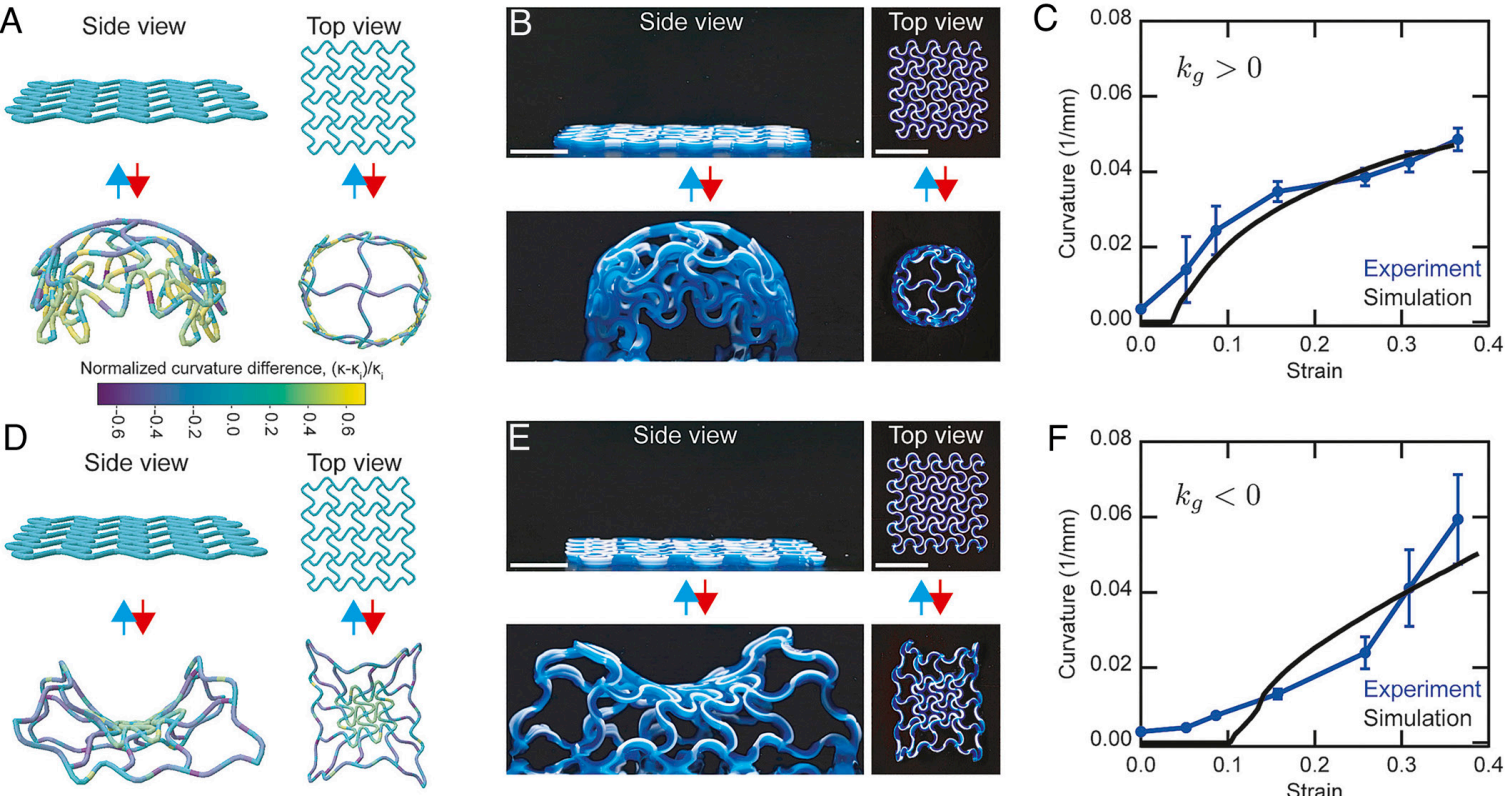
Active–passive lattices with heterogeneous shape morphing. (*A*) Simulated side and top views of a heterogeneous lattice with expanding filaments in the central unit cells and contracting filaments in the outer unit cells, shown in the initial flat state and after heating. The curvature change relative to the initial state is color-coded as $\left(\kappa -\kappa_{i}\right)/\kappa_{i}$. (*B*) Corresponding experimental side and top views before and after heating. [Scale bar: 10 mm (side view) and 25 mm (*Top* view).] (*C*) Evolution of the characteristic curvature ($\kappa =\sqrt{\left|k_{1}k_{2}\right|}$) as a function of strain for lattices morphing into positive Gaussian curvature ($\kappa_{g}=k_{1}k_{2}>0$). Simulation (black) and experiment (blue) are shown as mean ± SD (n = 3). (*D*) Simulated side and top views of a heterogeneous lattice with contracting filaments in the central unit cells and expanding filaments in the outer unit cells, shown in the initial flat state and after heating. (*E*) Corresponding experimental side and top views before and after heating. [Scale bar: 10 mm (side view) and 25 mm (*Top* view).] (*F*) Evolution of the characteristic curvature (κ) as a function of strain for lattices morphing into negative Gaussian curvature ($\kappa_{g}<0$). Simulation (black) and experiment (blue) are shown as mean ± SD (n = 3).

In our architected lattice composed of sinusoidal filaments, the effective elongation and contraction are programmed by the direction of bending: elongation corresponds to a reduction of bending curvature along the end-to-end direction, while contraction corresponds to an increase. This mismatch prevents purely in-plane deformation. Instead, the structure undergoes out-of-plane deformation, minimizing the energy associated with bending in the original end-to-end direction at the expense of increased transverse bending and twist (71). As a simple demonstration, two representative configurations are designed to realize opposite curvature behaviors. In the first, expansive filaments are positioned at the lattice center and contractive filaments along the perimeter. Both simulations and experiments show that this arrangement produced spherical morphologies with positive Gaussian curvature, as initially flat lattices rose into dome-like shapes upon actuation (Fig. 4 *A*–*C* and Movie S6). In contrast, inverting the pattern by placing contractive filaments at the center and expansive filaments at the perimeter yielded saddle-shaped morphologies with negative Gaussian curvature, again confirmed through simulations and experiments (Fig. 4 *D*–*F* and Movie S7). We note that the evolution of the characteristic curvature, defined as $\kappa \;=\sqrt{\left|k_{1}k_{2}\right|}$, where $k_{1}$ and $k_{2}$ are the principal curvatures, closely follows the measured changes in local edge lengths (*SI Appendix*, Fig. S18). Minor deviations arise from gravitational effects in the initially flat lattices, which require a finite temperature increase to overcome gravity and induce nonzero curvature. These gravitational effects are more pronounced in the negatively curved lattice due to its lower effective stiffness under vertical load, in contrast to the greater structural rigidity of domes with positive Gaussian curvature. Nonetheless, by spatially integrating expanding and contracting filaments within a single architecture, rather than restricting deformation to one mode to generate curvature (72), our approach enables reversible out-of-plane shape transformations of lattices with highly programmable morphologies.

Unlike prior demonstrations of lattices with programmable shape morphing, where bending arises in struts composed of multiple materials with mismatched properties (29, 73, 74), this current embodiment integrates active and passive materials within a single filament. Here, deformation follows from a prescribed natural curvature and twist field rather than from strain mismatch fixed by layered strut geometry, eliminating the need to pattern bilayer or trilayer architectures. Through rotational coprinting of active LCE and passive elastomer within one cross-section, we directly encode local natural curvature and twist along the filament centerline. This enables large, reversible in- and out-of-plane deformations by coupling the intrinsic actuation strain of LCEs with precisely positioned passive domains. Because the active and passive regions are integrated during printing, multimaterial lattices with programmable shape morphing are also realized in a single fabrication step, and the mechanical description correspondingly shifts from growing elastic surfaces (27, 28) to actively deforming elastic filaments. Ultimately, rotational multimaterial printing redefines where deformation is programmed by shifting it from patterned multilayer strut assemblies to a single, continuously programmed filament, thereby enabling precise control over lattice-scale deformation and its functional consequences.

**Fig. 5.**
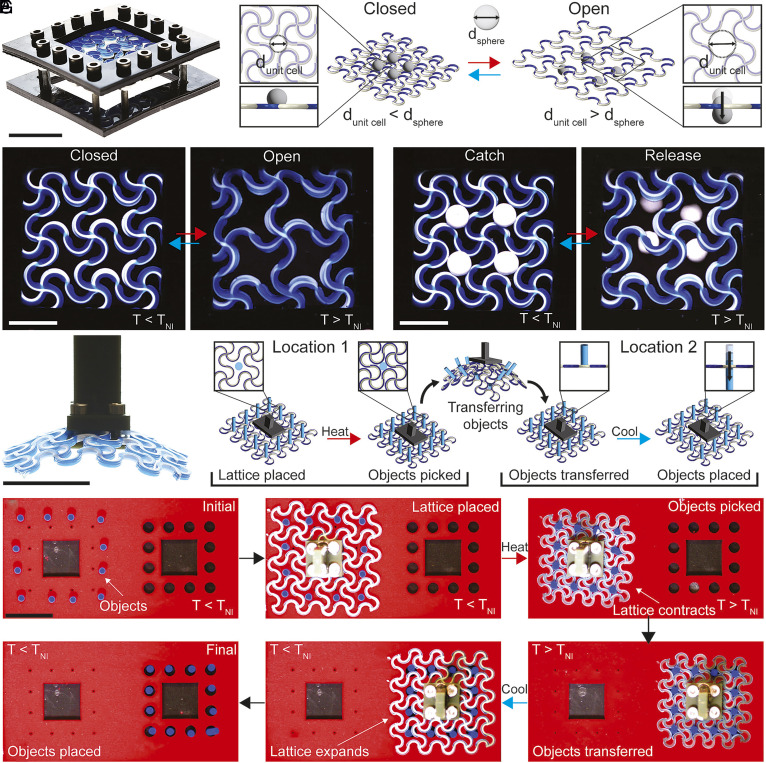
Active–passive lattices for filtering and gripping objects. (*A*) Expanding active–passive lattice filter mounted within an acrylic frame. (Scale bar: 25 mm.) (*B*) Schematic of the filter illustrating object capture at low temperature (closed state) and release at elevated temperature (opened state). (*C*) Filter transitioning from the closed to the opened state upon heating. (*D*) Active–passive lattice catching and releasing spheres. [Scale bar: 10 mm for (*C* and *D*).] (*E*) Pick-and-place gripper composed of a contracting lattice attached to an acrylic handle. (Scale bar: 25 mm.) (*F*) Schematic of the gripper illustrating object capture upon heating and release upon cooling. (*G*) Time-lapse images demonstrating pick-and-place multiple acrylic rods (3.5 mm diameter, 6 mm length). (Scale bar: 25 mm.)

This precise control of unit cell deformation enables soft robotic matter capable of manipulating objects in myriad ways. Active–passive lattices composed of unit cells programmed to expand upon heating can act as filters (Fig. 5 *A* and *B* and Movie S8). At low temperatures below $T_{\mathrm{NI}}$, the filter is in the closed state, where the unit cell aperture is smaller than the object diameter, enabling the lattice to catch the object. Upon heating above $T_{\mathrm{NI}}$, the lattice transitions to the open state, increasing the aperture size and releasing the object. This reversible closed–open transition enables on-demand capture and release. Furthermore, active–passive lattices can filter objects of varying size and geometry, such as spheres (Fig. 5 *C* and *D*). Beyond filtering, architected lattices can also transfer multiple objects simultaneously. For example, active–passive lattices programmed to contract upon heating can function as pick-and-place tools capable of transferring multiple objects between predefined locations (Fig. 5 *E*–*G* and Movie S9). As a simple demonstration, acrylic rods (3.5 mm in diameter and 6 mm in length) are transferred using a lattice-based gripper programmed to contract. Upon heating the lattice above $T_{\mathrm{NI}}$, the unit cells contract, leading to an aperture size smaller than the diameter of each rod, thereby gripping and securing the objects. Once secured, the lattice along with the objects is transferred to a second location with designated slots for placement. Cooling below $T_{\mathrm{NI}}$ causes the unit cells to expand, increasing the aperture and releasing the objects into their prescribed positions, where they remain even after the gripper is removed. Unlike most soft grippers reported to date, which typically manipulate a single object at a time, these lattices enable simultaneous pick-and-place of multiple objects.

## Conclusions

In summary, we establish a filament-centric strategy for programmable shape morphing by directly encoding intrinsic curvature and twist within multimaterial elastomeric filaments. Through rotational coprinting, we prescribe the natural curvature–twist field κ(s) of individual filaments, thereby achieving independent control over the full set of orientational degrees of freedom (i.e., the two spatially varying curvatures and twist). This strain gradient programming drives complex actuation via differential expansion of the constituent materials. Furthermore, by controlling rotation and print speed along prescribed paths, we architect lattices that exhibit heterogeneous shape morphing, including out-of-plane actuation, consistent with computational predictions based on DER theory. As demonstrations of function, these lattices operate as dynamic filters and multiobject grippers, illustrating how geometric control at the filament level translates into lattice-scale mechanical performance.

More broadly, encoding curvature and twist at the filament level allows us to program deformations geometrically without being tied to a specific material system. This allows the same design principles to extend beyond LCEs to other active materials, including hydrogels, shape-memory polymers, and dielectric elastomers, with material properties governing actuation amplitude, response time, and durability. Our numerical framework is likewise transferable across material classes through appropriate constitutive descriptions. Together, our generalizable theory, computation and digital fabrication framework enables the rapid design and printing of architected soft matter for adaptive materials, soft robotics, and deployable structures.

## Materials and Methods

### Materials.

Liquid crystal monomer, 1,4-bis-[4-(6-acryloyloxyhexyloxy) benzoyloxy]-2-methylbenzene (RM82), was purchased from Synthon chemicals. The photoinitiator, 2,2-dimethoxy-2-phenylacetophenone (Irgacure I-651), the chain extender, n-hexylamine, the inhibitor, butylated hydroxytoluene (BHT), and a two-part polydimethylsiloxane (PDMS) (Sylgard 184) elastomer were purchased from Fisher Scientific. Fumed silica, CAB-O-SIL EH-5 and CAB-O-SIL TS-720, were purchased from Cabot. Silicone oil was purchased from Oakwood Products (Lot No: 102516V20D). Ebecryl 8413 resin was purchased from Allnex. Pentaerythritol tetraacrylate was purchased from TCI Chemicals. The aliphatic urethane acrylate oligomer (CN9018) was purchased from Sartomer. Isodecyl acrylate was purchased from Sigma-Aldrich. Red and blue Silc Pig pigment dyes were purchased from Smooth-On.

### Ink Synthesis.

The active LCE ink was prepared via an aza–Michael addition reaction. RM82 and n-hexylamine were combined at a 1.4:1 molar ratio with 2 wt.% Irgacure I-651, 0.2 wt.% BHT, and 5 wt.% fumed silica. RM82, I-651, and BHT were weighed into a 20 mL vial and heated at 100 °C for 1 h to melt. n-Hexylamine was then added, and the mixture was homogenized using a heat gun and vortex mixing. Oligomerization was carried out at 75 °C for 18 h. After oligomerization, fumed silica (5 wt.%) was incorporated using a planetary mixer (FlackTek, Inc.) at 2,000 rpm for 10 min with 2 min rest intervals between mixing cycles. The ink was transferred to a 10 cc UV/light-block amber syringe (Nordson EFD) and degassed by centrifugation at 3,000 rpm for 3 min (Heraeus Megafuge 8) to remove trapped air.

The passive elastomer ink was prepared from a mixture of 41.1 wt.% CN9018, 41.1 wt.% isodecyl acrylate, 0.8 wt.% Irgacure I-651, and 17 wt.% fumed silica (CAB-O-SIL TS-720). All components except the silica were first combined in a disposable polypropylene mixing cup. Fumed silica was added in two portions: half was incorporated and mixed at 2,000 rpm for 10 min (2 min mixing intervals with rest periods) using a planetary mixer (FlackTek, Inc.), followed by addition of the remaining silica and a second mixing cycle. The mixture was manually reintegrated and subjected to a third mixing cycle to ensure homogeneity. Blue pigment dye (0.5 wt.%) was added prior to the final mixing step. The ink was transferred to a 30 cc UV/light-block amber syringe (Nordson EFD) and degassed by centrifugation at 2,500 rpm for 10 min (Avanti J-25 I) to remove trapped air.

To increase the elastic modulus of the passive elastomer, it was blended with a stiffer elastomer ink at ratios of 1:10 and 1:4 (stiffer ink: passive ink). The stiffer ink was prepared from Ebecryl 8413 resin and pentaerythritol tetraacrylate (1:1 by weight) with 10 wt.% fumed silica (CAB-O-SIL EH-5) and 4 wt.% Irgacure I-651, mixed using the same protocol described above.

### Rotational Multimaterial 3D Printing.

Printing was performed following a previously reported protocol (56) with minor modifications. Separate syringes containing the active and passive elastomer inks were mounted onto a custom dual-channel nozzle fabricated using a D4K digital light processing (DLP) printer from a rigid photopolymer resin (*SI Appendix*, Fig. S1). The nozzle comprised two semicircular internal channels that merged at a 1 mm-diameter outlet to enable coextrusion. Each syringe was connected to an independent digital pressure controller (PCD-100PSIG-D, Alicat Scientific) through pneumatic couplings, enabling synchronized pressure control during print motion.

Filaments, printed with or without nozzle rotation, were deposited onto cleaned glass substrates and subsequently UV-cured under an argon atmosphere for 10 min per side. UV curing was performed using a UV lamp (Omnicure Series 2000, 200 W, 320 to 500 nm; Lumen Dynamics Inc.) housed in a UV box. Lattices were printed layer-by-layer on glass substrates and UV-cured after each layer. Following completion of the print, the entire lattice was UV-cured for an additional 10 min per side under argon to ensure complete crosslinking. After curing, filaments and lattices were carefully removed from the substrates using a fresh razor blade.

### Thermal Characterization.

Differential scanning calorimetry (DSC; Discovery DSC 250, TA Instruments) was used to determine the nematic-to-isotropic transition temperature ($T_{\mathrm{NI}}$) of the active LCE ink. Samples (≥5 mg) were sealed in aluminum pans and subjected to a heating–cooling–reheating cycle from room temperature to 200 °C, cooling to −50 °C, and reheating to 200 °C at 10 °C min^−1^. $T_{\mathrm{NI}}$ was defined as the peak of the melting endotherm obtained from the second heating cycle and determined using the enthalpy-of-melt analysis tool in TA TRIOS software. Measurements were performed in triplicate.

The glass transition temperature ($T_{g}$) of the crosslinked active and passive elastomers was measured using DSC under similar conditions. Samples (≥4 mg) were heated to 200 °C, cooled to −90 °C, and reheated to 200 °C. Heating was performed at 10 °C min^−1^ and cooling at 5 °C min^−1^. A slower cooling rate was used to reduce kinetic undercooling and improve resolution of the glass-transition features. $T_{g}$ was determined from the second heating cycle using the glass-transition analysis tool in TA TRIOS software. All measurements were conducted in triplicate.

### Actuation Measurements.

Actuation of bulk materials and architected multimaterial filaments and lattices was characterized by immersing samples in a temperature-controlled silicone oil bath. Images and videos were recorded using a uEye camera (Imaging Development Systems Inc.), while temperature was monitored with a thermocouple (Fluke T3000). The normalized lengths of bulk active and passive elastomer samples were measured during heating from 25 °C to 150 °C. Bilayer curvatures (active/passive), printed at varying speeds without angular rotation, were measured during heating from 25 °C to 175 °C.

For architected filaments printed at varying angular rotation rates and a constant translation velocity of 3 mm s^−1^, the end-to-end normalized length change was determined by imaging at 25 °C, heating to 175 °C, and reimaging. Area changes in homogeneous architected lattices were quantified during heating and cooling between 25 °C and 175 °C. Thermal cycling tests were conducted by alternating samples between oil baths maintained at 25 °C and 175 °C.

Heterogeneous lattices were imaged using dual cameras (uEye for top view and Canon Rebel EOS Ti2 for side view). For all samples, images were captured at 25 °C prior to heating to 175 °C. All measurements were performed on three independently prepared samples and analyzed using ImageJ (version 1.53t). Cyclic actuation of Janus filaments printed without rotation was evaluated over 100 cycles by alternating immersion between oil baths at 25 °C and 175 °C, with imaging performed at 25 °C before each heating step.

### Rheological and Mechanical Characterization.

The rheological properties of the active and passive elastomer inks were measured using a rheometer (TA Instruments HR-20) equipped with a 20 mm steel Peltier parallel-plate geometry and a 300 µm gap. After loading, samples were heated to 125 °C and held for 300 s to erase thermal history, then cooled to 26 °C and equilibrated for 500 s prior to measurement. Viscosity was measured as a function of shear rate using a logarithmic sweep from 0.01 s^−1^ to 1,000 s^−1^. All measurements were performed on three independently prepared samples.

Tensile properties of bulk elastomers and active–passive multimaterial filaments were measured using the same instrument equipped with tensile grips. Printed filaments (~600 µm diameter, 30 mm length) were strained at 100 µm s^−1^ until failure. Measurements were conducted on three samples per condition.

Bilayer adhesion tests were performed using the hybrid rheometer equipped with tensile grips. Samples were prepared by injection molding the active and passive elastomer inks into rectangular specimens incorporating a 1 mm-wide interfacial gap to provide an engineered delaminated region for gripping. The two elastomers were crosslinked simultaneously. During testing, the active elastomer region was clamped in the lower grip and the passive elastomer free end in the upper grip, followed by tensile loading at 100 µm s^−1^ until failure. All adhesion measurements were performed in triplicate.

### WAXS.

Laboratory X-ray measurements were performed in transmission mode using a Xeuss 3.0 beamline (Xenocs Inc.). Scattering patterns were collected at 8.04 keV with a beam spot size of ~1.4 mm and an exposure time of 300 s using a Pilatus 300 K detector (Dectris). A single stitched scattering pattern was constructed from measurements acquired at multiple detector positions to eliminate detector gaps. Background subtraction was performed using empty-air measurements for pure active LCE samples and pure passive elastomer measurements for active–passive composites. Sample-to-detector distance calibration was carried out using a LaB_6_ diffraction standard.

Scalar orientational order parameters, $S=<P_{2}(\cos\chi )>$, were determined by integrating the mesogen scattering peak intensity over the range $q=0.65-2.5\;{Å}^{-1}$ as a function of azimuthal angle and fitting the resulting distribution using the Kratky method (75). Data reduction and stitching were performed using custom Python scripts based on pyFAI (76).

The spatial phase behavior of printed fibers was further characterized by transmission WAXS at the Soft Matter Interfaces beamline (12-ID SMI) at the National Synchrotron Light Source II (NSLS-II), Brookhaven National Laboratory (77). Scattering patterns were recorded using a Pilatus 900 K-W detector with an incident X-ray energy of 16.1 keV. The beam dimensions (full width at half maximum) were 25 µm (horizontal) × 2.5 µm (vertical), and the beam was aligned along the x-axis, perpendicular to the nematic director. Measurements were conducted under ultra-high vacuum at room temperature. Order parameters were calculated as described above.

### Microscopy.

Scanning electron microscopy (Zeiss Gemini 360 FE-SEM) (SEM) was used to image cross-sections of the filaments prepared through nitrogen freeze-fracture. Liquid nitrogen was placed in a Styrofoam container and held under vacuum for approximately 5 min to bring its temperature below the glass transition temperature of both elastomers. The fiber samples were then immediately submerged in liquid nitrogen. After 2 to 5 min, the fibers were fractured by snapping them in half with two self-closing tweezers. The fractured surfaces were mounted onto 90° stubs (Ted Pella) using conductive carbon tape. A 10 nm Pt/Pd conductive coating was subsequently sputtered onto the mounted samples using an EMS150T S sputter coater. Bright field microscopy (Axio Observer, Zeiss) was used to image filaments. Images were analyzed using ImageJ software (version 1.53t).

### Active Filter.

The filter assembly consisted of a heterogeneous lattice designed to contract along its perimeter while expanding in the center, thereby minimizing out-of-plane deformation under edge constraints. The lattice was mechanically secured to a laser-cut acrylic frame using 3 mm screws to attach the lattice to the top plate and 16 mm screws to fasten the top plate to the bottom support plate. The assembled structure was immersed in a silicone oil bath and imaged from above using a uEye camera while temperature was monitored with a thermocouple. To demonstrate filtering functionality, grinding media spheres (5 mm diameter, 450 mg; Inframat Advanced Materials) and M4 bolts (7 mm end-to-end length, 650 mg) were used as representative objects for the closed and open filter states.

### Pick and Place.

The gripper consisted of a contracting lattice attached to a laser-cut acrylic square base using M3 screws and bolts. The PDMS base was fabricated by replica molding from a rigid positive master printed using a D4K DLP printer. The printed master (120 mm × 75 mm × 6 mm) contained raised square platforms (19 mm × 19 mm × 3 mm), cylindrical posts (6 mm diameter, 3 mm height) defining the placement slots, and an array of raised alignment markers. A PDMS prepolymer mixture (Sylgard 184), dyed with red Silc Pig pigment, was poured over the printed master and cured at 75 °C for 12 h. After curing, the PDMS base was peeled from the master, yielding a negative replica with recessed square features, cylindrical cavities, and alignment dimples. Acrylic rods were initially positioned on the PDMS surface using the alignment dimples as fiducial markers. The gripper was then placed over the rods, and the assembly was heated to 175 °C in a silicone oil bath (temperature monitored by thermocouple) to contract the lattice and grasp the rods. The gripper carrying the rods was transferred to the target region containing the recessed cavities, and the bath was cooled to 50 °C to release the rods as the lattice expanded. Video was recorded from the top view using a uEye camera.

### Discrete Element Rod (DER) Model.

We employed the DER model to efficiently compute the minimal elastic energy configurations (58, 78). The filament’s centerline is discretized into a set of N vertices $r_{s}$ connected by edges $e_{s}=r_{s+1}-r_{s}$, where the previously continuous arc length parameter now s becomes an integer index along the filament’s centerline. Discrete curvature and twist are defined at each interior vertex s.

With the material frame {$d_{1,s},d_{2,s},d_{3,s}$} defined at each interior vertex s, the curvature binormal (which captures bending) is then computed as

$${\left(k_{b}\right)}_{s}=\frac{2e_{s-1}\times e_{s}}{\left|e_{s-1}\right|\left|e_{s}\right|+e_{s-1}\cdot e_{s}}.$$

The components $k_{1,s}$ and $k_{2,s}$ are the projections of $(k_{b}{)}_{s}$ onto the local basis vectors $d_{1,s}$ and $d_{2,s}$, while the discrete twist $\tau_{s}$ measures rotation of the material frame between adjacent edges. The continuous elastic energy integral is approximated by a sum over the vertices. The energy at each vertex s is weighted by the inverse of the Voronoi length $-l_{s}=\left(\left|e_{s-1}\right|+\left|e_{s}\right|\right)/2$. The total discrete elastic energy is then given by

$$\begin{array}{c} E_{\text{discrete}}=\sum_{s=1}^{N-1}\frac{1}{2{\bar{l}}_{s}}\\ \;\left[B\left({\left(\kappa_{1,s}-{\bar{\kappa }}_{1,s}\right)}^{2}+{\left(\kappa_{2,s}-{\bar{\kappa }}_{2,s}\right)}^{2}\right)+C{\left(\tau_{s}-{\bar{\tau }}_{s}\right)}^{2}\right], \end{array}$$

where B and C are the bending and torsional rigidities, ${\bar{l}}_{s}$ is the Voronoi length and ${\bar{\kappa }}_{1,s}$, ${\bar{\kappa }}_{2,s}$, and ${\bar{\tau }}_{s}$ are the natural curvatures and twist, respectively. The equilibrium configuration corresponds to the set of vertex positions $\left\{r_{s}\right\}$ that minimize $E_{\text{discrete}}$, found using a standard gradient descent algorithm implemented in the open-source package disMech (78).

### Numerical Simulation.

We model the architected lattice as a discrete graph consisting of 25 branch nodes—corresponding to junctions where sinusoidal filaments meet—and 40 connecting edges. Each edge is discretized further into 10 ghost nodes to resolve the sinusoidal geometry, resulting in a total of 425 nodes (*SI Appendix*, Fig. S13 *A* and *B*).

#### Parameterizing the shape-morphing lattice in numerical simulations.

First, we consider a simple square grid with n points on each side. The grid has a total of $N=n^{2}$ points, which we call vertices. We assign each one a unique number (index) from 0 to N-1. To find the index k for a vertex at row i and column j, we use row-major order: $k=\left(n\times i\right)+j$. If the distance between any two adjacent points is l, the spatial coordinate $v_{k}$ for the vertex at $\left(i,j\right)$ is given by $v_{i,j}=\left(l\cdot i,l\cdot j\right)$.

#### Creating wavy edges.

Next, we replace the straight lines (edges) connecting the vertices with curves. To do this, we add a series of new points, called *ghost nodes*, along the path of each original edge. These ghost nodes are not placed in a straight line; they are offset to form a sine wave, making the edge appear wavy.

#### The formula for ghost nodes.

To calculate the exact position of each ghost node along an edge, we use the following method. Consider a straight edge from a starting vertex $v_{\text{start}}$ to an ending vertex $v_{\text{end}}$. To place $n_{g}$ ghost nodes along it, we define: 1) the unit vector T pointing from $v_{\text{start}}$ to $v_{\text{end}}$ which is the direction of the straight edge and 2) the unit vector N perpendicular to T which is the direction of the wave’s oscillation, and 3) the amplitude, or maximum height, of the wave, A. The position of the s-th ghost node (where s is an integer from 1 to $n_{g}$) is a combination of moving along the straight edge and then adding a perpendicular offset for the wave:

$$v_{\text{ghost}}\left(s\right)=\underset{\text{Position along straight line}}{\underbrace{v_{\text{start}}+T\left(\frac{s\cdot l}{n_{g}+1}\right)}}+\underset{\text{Perpendicular wave offset}}{\underbrace{N\;A\text{sin}\left(\frac{2\pi s}{n_{g}+1}\right)}}.$$

#### Gradual increase in natural curvature amplitude.

We simulate the continuous transformation from a flat sheet to the final 3D object by incrementally increasing the parameter x from 0 to 0.75, so that $c_{s}=1+x$ ranges from 1 to 1.75 (for straightening edges) and $c_{c}=1-x$ from 1 to 0.25 (for curling edges) where *c*_s_ and *c*_c_ are the ratio of natural curvatures between before and after thermal activation in each filament, corresponding to straightening and curling edges, respectively. At each step, the structure’s stable equilibrium shape is found by minimizing its total elastic energy, initialized from the solution of the previous step.

## Supplementary Material

Appendix 01 (PDF)

Movie S1. Rotational 3D printing of active-passive filaments at a constant print speed of 3 mm s^-1^ while the rotation rate (ω∗) systematically increases from 0 to 0.5.

Movie S2. Active-passive filaments printed at 3 mm s^−1^ with varying rotation rates ((ω∗ = 0, 0.06, 0.13, 0.25, 0.5, 1.0, and 2.0) exhibit distinct bending and twisting behaviors upon heating.

Movie S3. Rotational 3D printing of an active-passive lattice at a print speed of 3 mm s^-1^.

Movie S4. Shape morphing response of an active-passive (expanding) lattice in which the active elastomer positioned on the outer radius of curvature. The lattice expands upon heating and returns to original shape upon cooling.

Movie S5. Shape morphing response of an active-passive (contracting) lattice in which the active elastomer positioned on the inner radius of curvature. The lattice contracts upon heating and returns to the original shape upon cooling.

Movie S6. Shape morphing response of a heterogeneous active-passive lattice (positive gaussian curvature), in which the central unit cells expand, while the outer unit cells contract. When submerged in heated silicone oil, the initially flat lattice morphs into a dome. Simulations confirm the observed deformation.

Movie S7. Shape morphing response of a heterogeneous active-passive lattice (negative gaussian curvature), in which the central unit cells contract, while the outer unit cells expand. Upon heating in silicone oil, the initially flat lattice morphs into a saddle-shaped configuration with negative gaussian curvature, consistent with simulations.

Movie S8. Active-passive lattice filter expands upon heating when submerged in silicone oil allowing 3D objects (e.g., 5-mm spheres or hexagonal nuts) to either be trapped (closed unit cells) or pass through (opened unit cells).

Movie S9. Pick-and-place of multiple objects using an active-passive lattice. A contracting lattice is placed in a silicone oil bath containing an array of 3.5-mm-diameter acrylic rods. Upon heating, the unit cells contract and grip the rods. The rod-filled lattice is then transferred to a new location containing 6-mm-diameter holes. Upon cooling, the unit cells expand and release each rod into its corresponding hole.

## Data Availability

The simulation code for the shape-morphing network is publicly available at Github (79.) Study data are included in the article and/or supporting information.
