# Supplementary material for: Rotational 3D printing of active–passive filaments and lattices with programmable shape morphing
Source: Proc Natl Acad Sci U S A. 2026 Apr 22;123(17):e2537250123. doi: 10.1073/pnas.2537250123 (PMC13123922; doi:10.1073/pnas.2537250123)
Supplement: Supplementary file 1 — Appendix 01 (PDF) [file pnas.2537250123.sapp.pdf]

PNAS

Supporting Information for

Rotational 3D printing of active-passive filaments and lattices with programmable shape morphing

Mustafa K. Abdelrahman[1], Jackson K. Wilt[1], Yeonsu Jung[1], Rodrigo Telles[1], Gurminder K. Paink[1], Natalie M. Larson[1,2], Joanna Aizenberg[1,3], L. Mahadevan[1,4,5*], and Jennifer A. Lewis[1,6*]

[1]Harvard John A. Paulson School of Engineering and Applied Sciences, Harvard University, Cambridge, MA, USA

[2]Department of Mechanical Engineering, Stanford University, Stanford, CA, USA

[3]Department of Chemistry and Chemical Biology, Harvard University, Cambridge, MA, USA

[4]Department of Physics, Harvard University, Cambridge, MA, USA

[5]Department of Organismic and Evolutionary Biology, Harvard University, Cambridge, MA, USA

[6]Wyss Institute for Biologically Inspired Engineering, Harvard University, Cambridge, MA, USA

*Corresponding Authors: L. Mahadevan and Jennifer A. Lewis

Email: lmahadev@g.harvard.edu; jalewis@seas.harvard.edu

This PDF file includes:

Figures S1 to S18
Legends for Movies S1 to S9

Other supporting materials for this manuscript include the following:

Movies S1 to S9

Figures

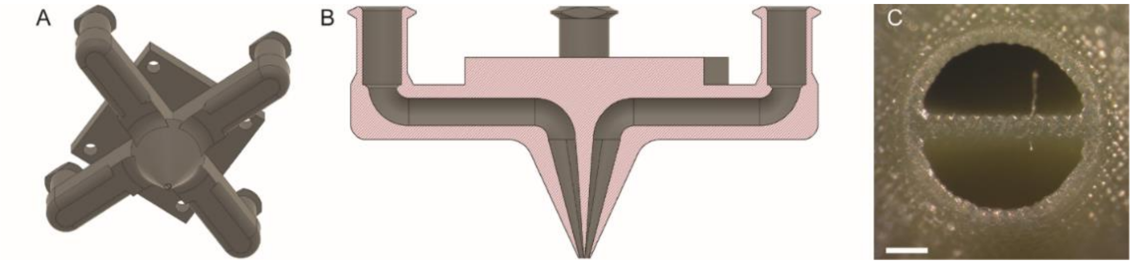

Fig. S1. (A) Custom-designed nozzle used for rotational multi-material 3D printing. (B) Cross-section of the custom nozzle used for rotational multi-material 3D printing depicting the two channels where the active and passive elastomer inks flow through. (C) Image of nozzle opening showing two separate channels for co-extrusion of active-passive elastomer inks. Scale bar: 250 μm.

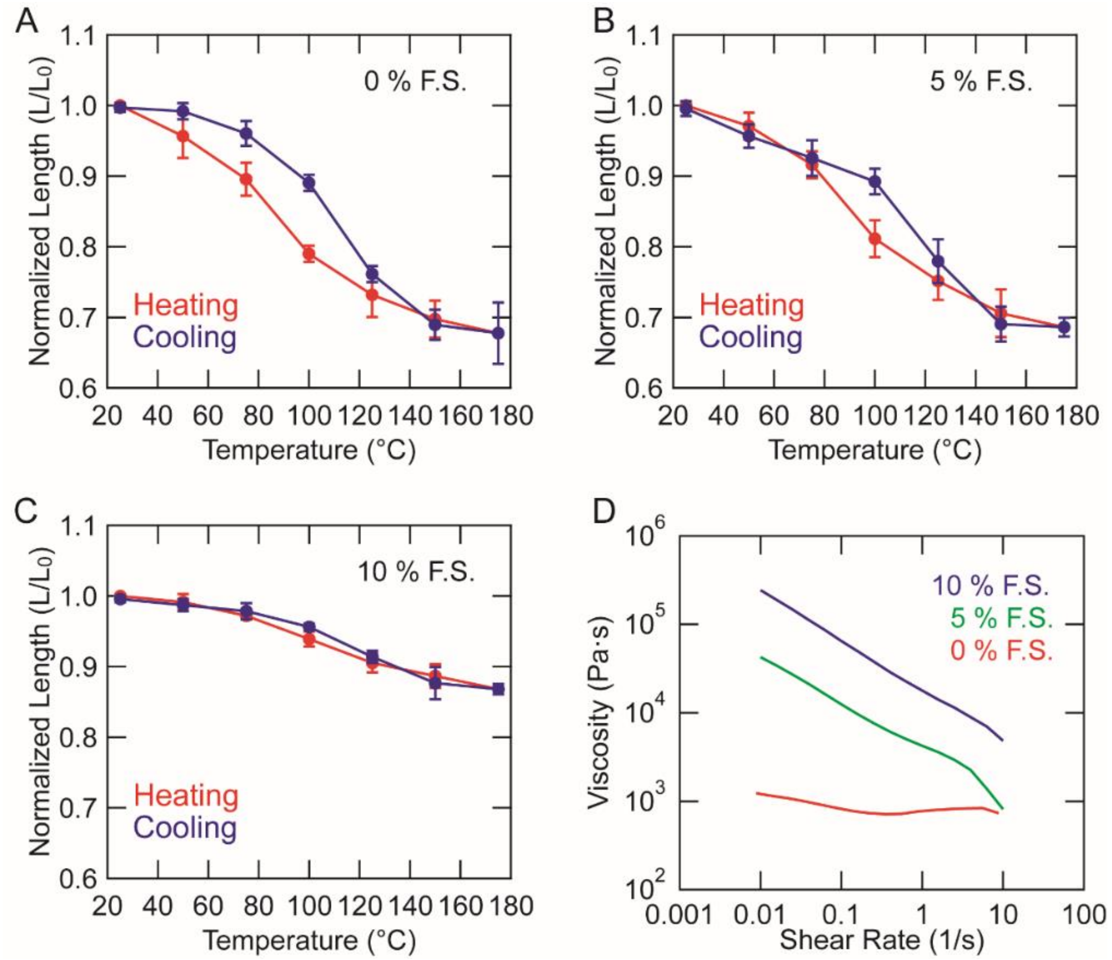

Fig. S2. Normalized length plotted as a function of temperature for the pure LCE (active) elastomer with (A) 0 wt. % fumed silica, (B) 5 wt. % fumed silica, and (C) 10 wt. % fumed silica loading demonstrating similar thermal actuation between the 0 wt. % loading and the 5 wt. % loading. Note, the actuation of the 10 wt. % loaded sample demonstrates less deformation. The red curves represent heating while the blue curves represent cooling. Data are shown as mean ± s.d. (n = 3). (D) Representative viscosity plotted as a function of shear rate for LCE inks with 10 wt.% fumed silica loading (blue curve), 5 wt.% fumed silica loading (green curve), and 0 wt.% fumed silica loading (red curve), demonstrating increasing viscosity and shear thinning response as fumed silica loading increases.

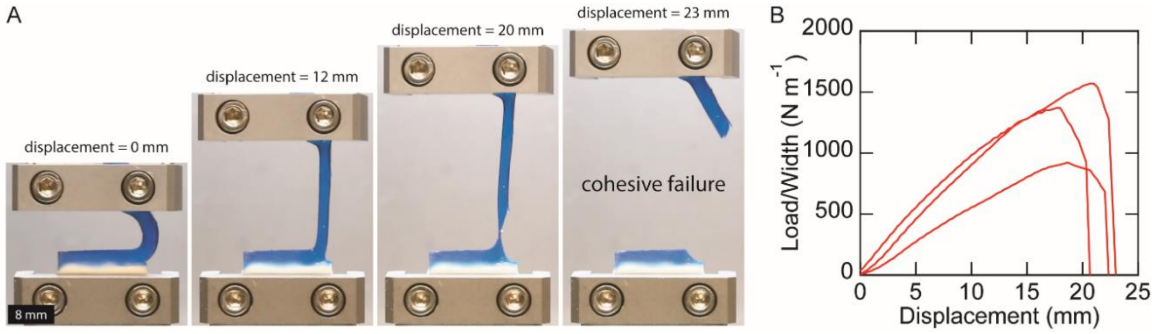

Fig. S3. (A)Time-lapse images of a 90° peel test demonstrating cohesive failure of the passive region within a Janus (active-passive) sample. (B) Load/width (N m^{-1}) plotted as a function of displacement for a 90° peel test for three samples. All plots are shown. Peak adhesion strength = 1289 ± 334 N m^{-1} (n = 3).

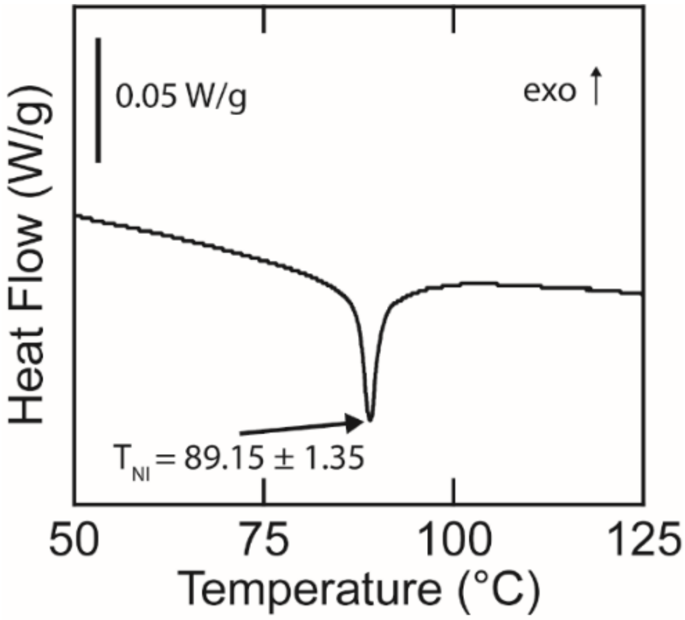

Fig. S4. Representative DSC thermogram showing heat flow as a function of temperature of the active elastomer ink measuring the T_{NI}. The T_{NI} = 89.15 ± 1.35 °C (n = 3).

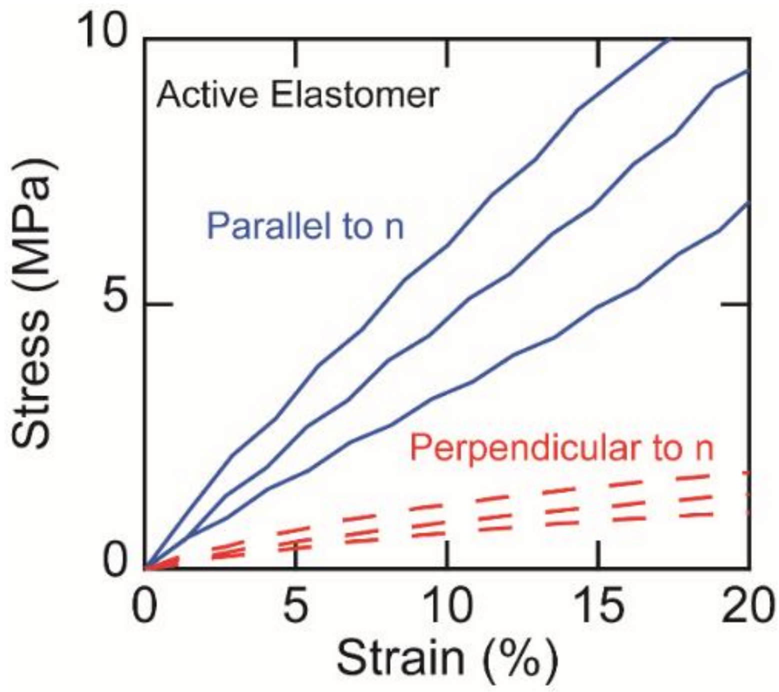

Fig. S5. Stress plotted as a function of strain of the pure LCE (active) elastomer strained either in the direction parallel to the n director (blue) or perpendicular to the n director (red). Nematic director is parallel to the print path. Average elastic modulus along the director is 44.84 ± 8.7 MPa and the mean elastic modulus perpendicular to the director is 13.46 ± 2.7 MPa. All three plots are shown.

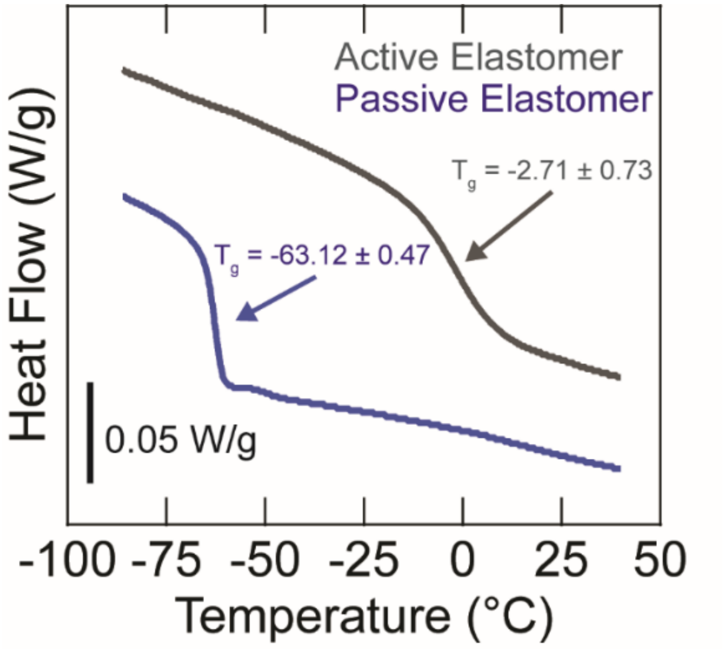

Fig. S6. Representative DSC thermograms showing heat flow as a function of temperature for the pure, crosslinked LCE (active, gray curve) elastomer and the pure, crosslinked acrylate-based (passive, blue curve) elastomer. The LCE (gray) exhibits a glass transition temperature of −2.71 ± 0.73 °C, while the passive elastomer (blue) exhibits a glass transition temperature of −63.12 ± 0.47 °C (n = 3).

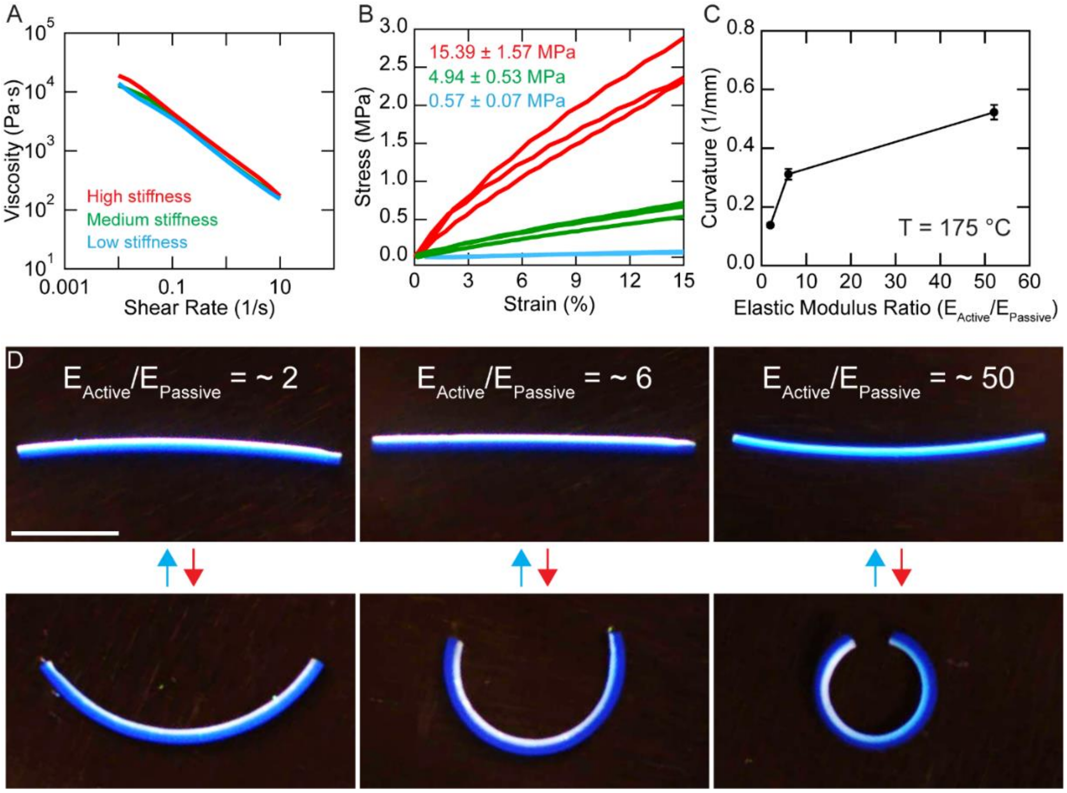

Fig. S7. (A) Representative viscosity plotted as a function of shear rate demonstrating shear thinning of all passive elastomer inks. (B) Stress plotted as a function of strain for the three passive materials, demonstrating a systematic decrease in elastic modulus from high-to-low stiffness formulations. (C) Maximum curvature plotted as a function of elastic modulus ratio ($E_{Active}/E_{Passive}$) demonstrating increased curvature with increasing modulus contrast. Data are shown as mean ± s.d. (n = 3). (D) Representative images of filaments printed with no rotation at a print speed of 3 mm s^{-1} fabricated with passive layers of decreasing stiffness. Top images show filaments in the unactuated state, while bottom images show their heated state, highlighting progressive increase in bending curvature as the passive layer softens.

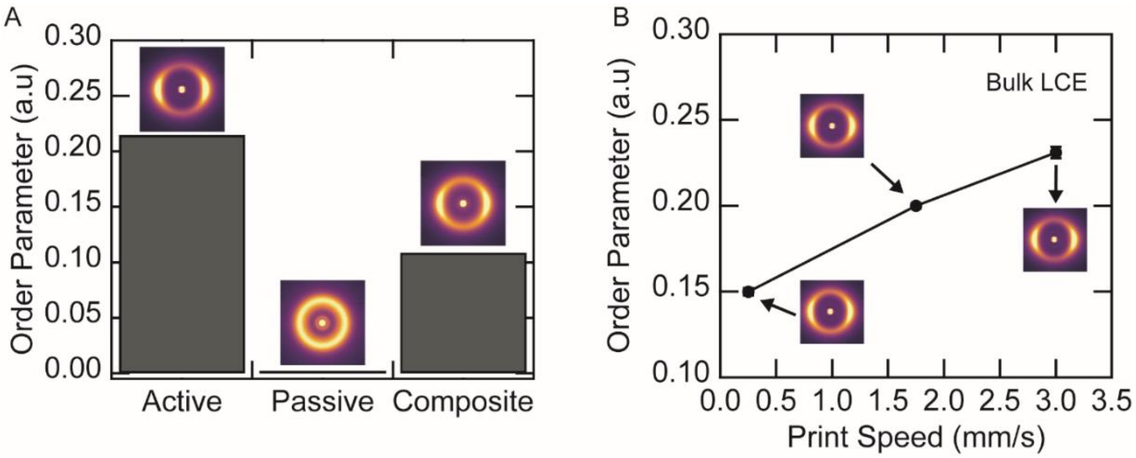

Fig. S8. (A) Order parameter (S) obtained for the pure LCE (active) elastomer, the pure acrylate-based (passive) elastomer, and the Janus (active-passive) elastomer filaments. (B) Order parameter (S) plotted as a function of print speed of the pure LCE (active) elastomer. Data are shown as mean ± s.d. (n = 3).

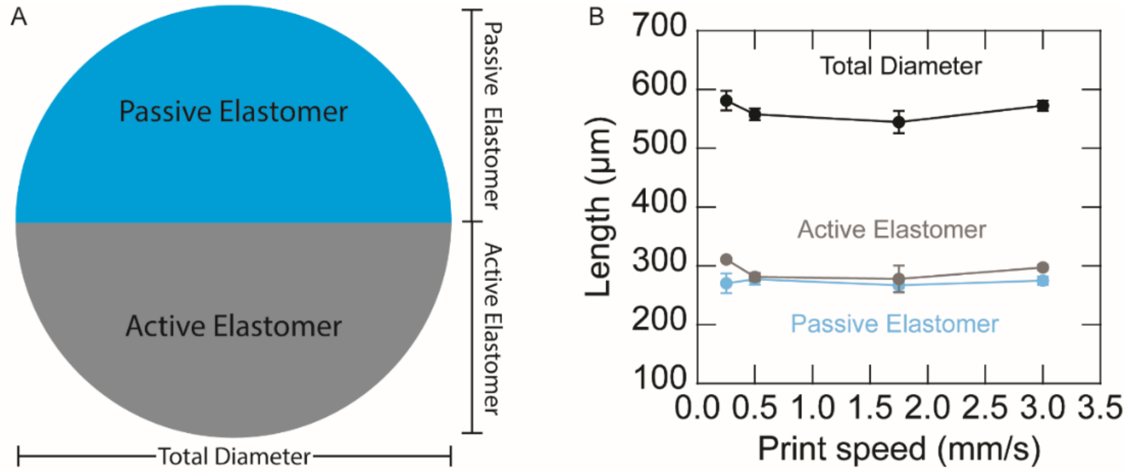

Fig. S9. (A) Schematic view of the active-passive filament cross-section. (B) Filament diameter and active/passive elastomer segment lengths as a function of print speed, measured from SEM images. Data are shown as mean ± s.d. (n = 3).

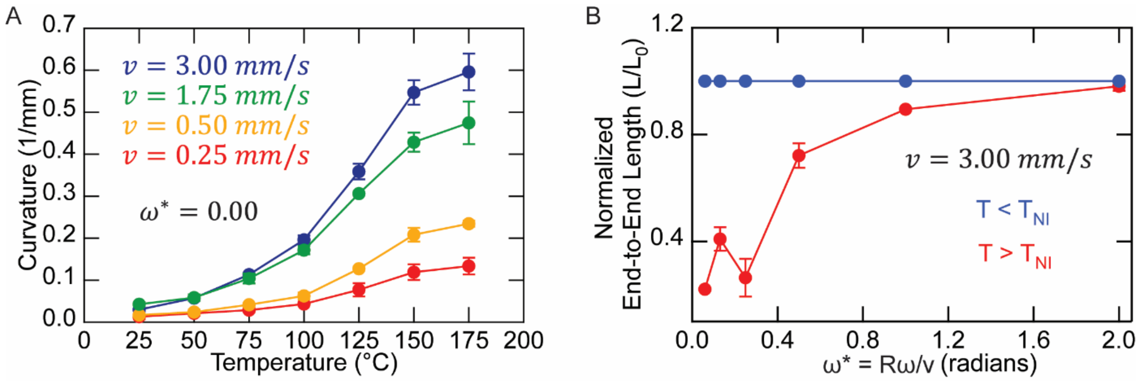

Fig. S10. (A) Curvature as a function of temperature for Janus (active-passive) filaments printed at varying speeds at a constant $\boldsymbol{\omega}^* = \mathbf{0}$. (B) Normalized end-to-end filament length plotted as a function of $\boldsymbol{\omega}^*$ for active-passive filaments printed at a constant print speed of 3 mm s^{-1} below T_{NI} (blue) and above T_{NI} (red). Data are shown as mean ± s.d. (n = 3).

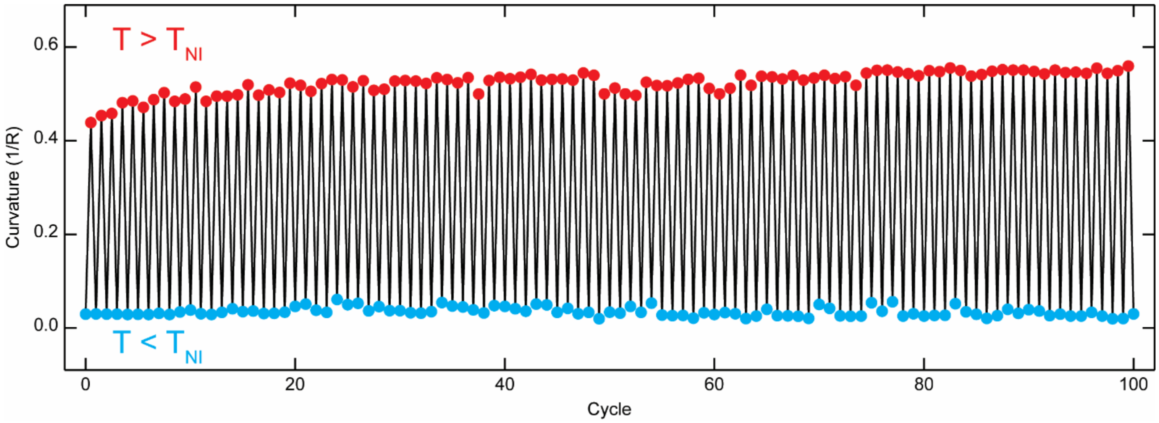

Fig. S11. Curvature plotted against cycle number of filament printed with no rotation upon cyclic heating to 175 °C (red) and cooling to 25 °C (blue), demonstrating reversibility.

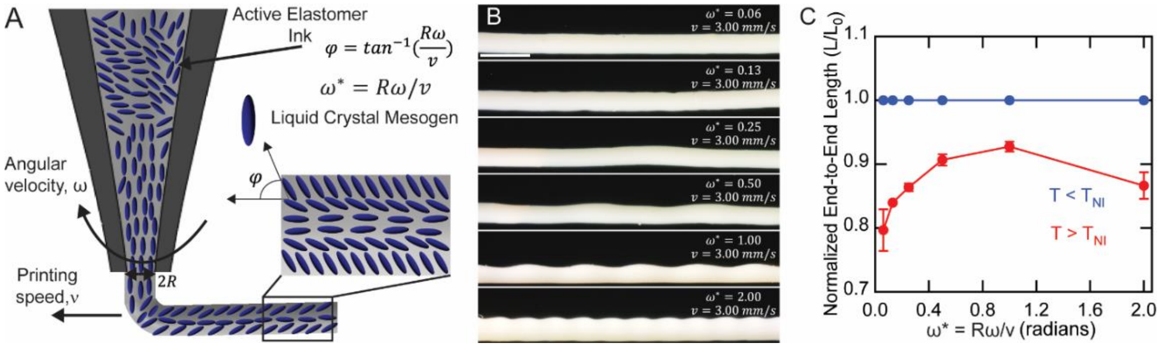

Fig. S12. (A) Schematic illustration of rotational printing of a pure LCE ink. (B) Optical images of pure LCE filaments printed at different rotation rates $\boldsymbol{\omega}^*$ at a constant print speed of 3.00 mm s^{-1}. Scale bar: 1 mm. (C) Normalized end-to-end filament length as a function of $\boldsymbol{\omega}^*$ at a constant print speed of 3 mm s^{-1} for pure LCE filaments below T_{NI} (blue) and above T_{NI} (red). Data are shown as mean ± s.d. (n = 3).

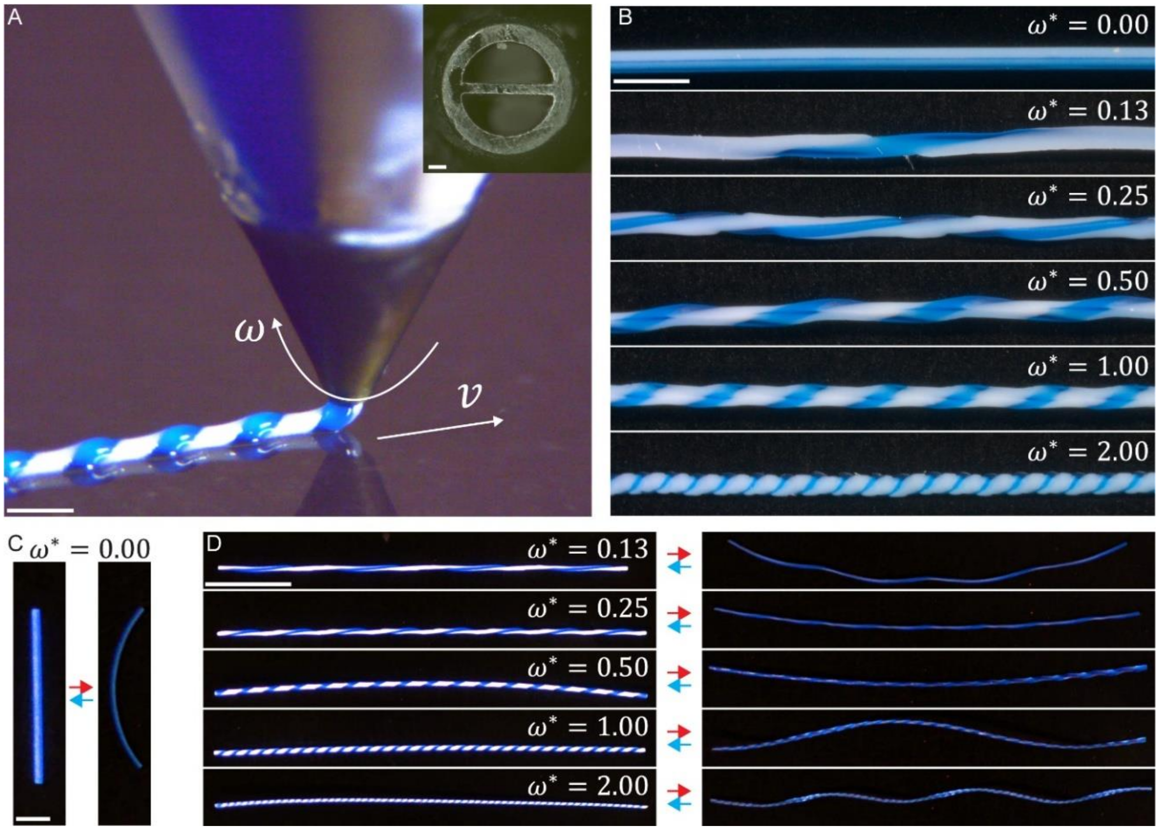

Fig. S13. (A) Image of ink co-extrusion through a 500 µm diameter nozzle during rotational printing. Scale bar: 1 mm, inset scale bar: 100 µm. (B) Optical images of architected filaments printed at different values of $\boldsymbol{\omega}^*$ at a constant velocity of 0.5 mm s^{-1}. Scale bar: 1 mm. (C) Representative images of a Janus filament printed at a constant $\boldsymbol{\omega}^* = \mathbf{0}$ and at a constant velocity of 0.5 mm s^{-1} in their initial state and heated state. Scale bar: 2 mm. (D) Representative images of architected composite filaments printed at varying $\boldsymbol{\omega}^*$ at a constant velocity of 0.5 mm s^{-1}. Scale bar: 10 mm.

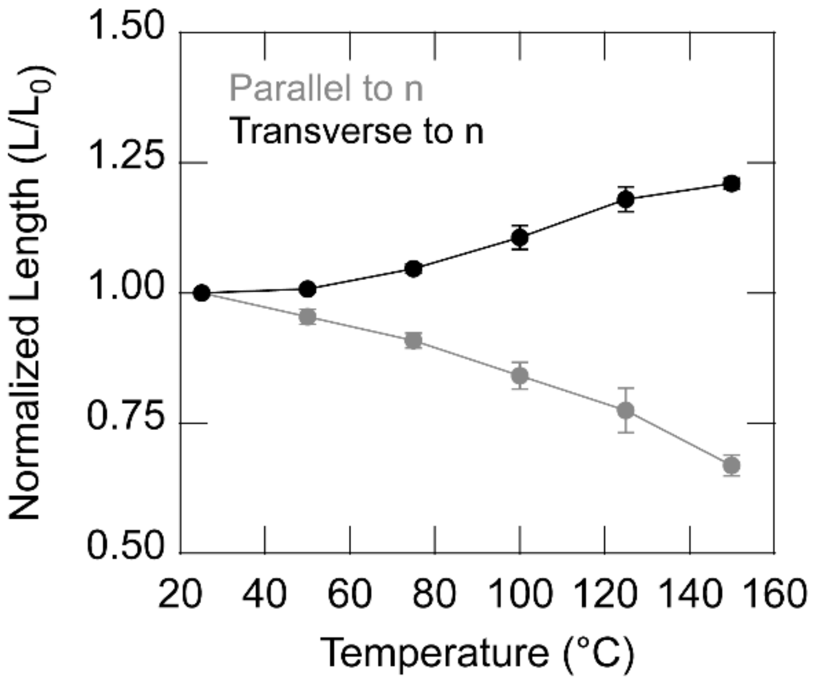

Fig. S14. Normalized length of printed, pure active LCE, measured parallel (grey) and transverse (black) to the nematic director (n). Data are shown as mean ± s.d. (n = 3).

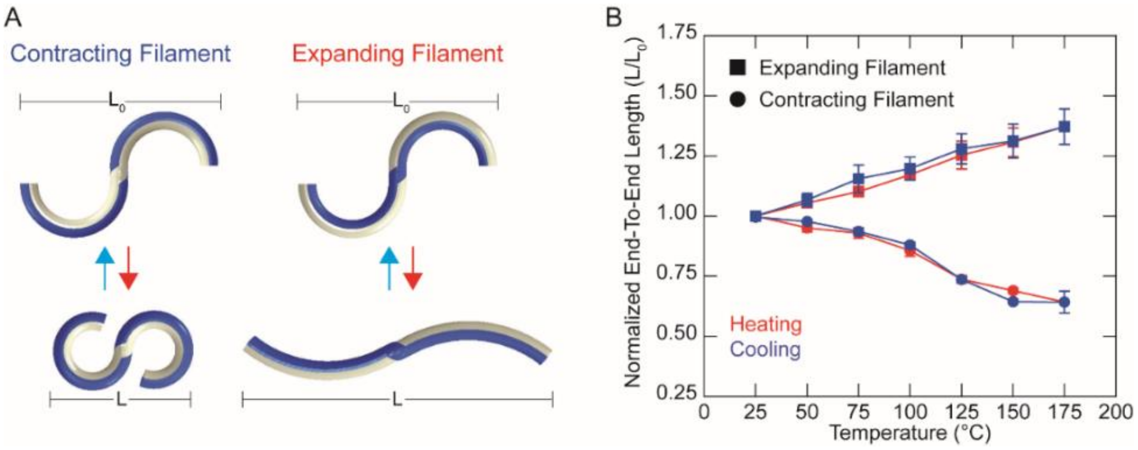

Fig. S15. (A) Schematic views of active-passive sinusoidal filaments demonstrating their respective increase or decrease in length upon thermal cycling above T_{NI}. (B) Normalized end-to-end filament length as a function of temperature. Expanding filaments are depicted as squares and contracting filaments are depicted as circles. The red curves represent heating while the blue curves represent cooling. Data are shown as mean ± s.d. (n = 3).

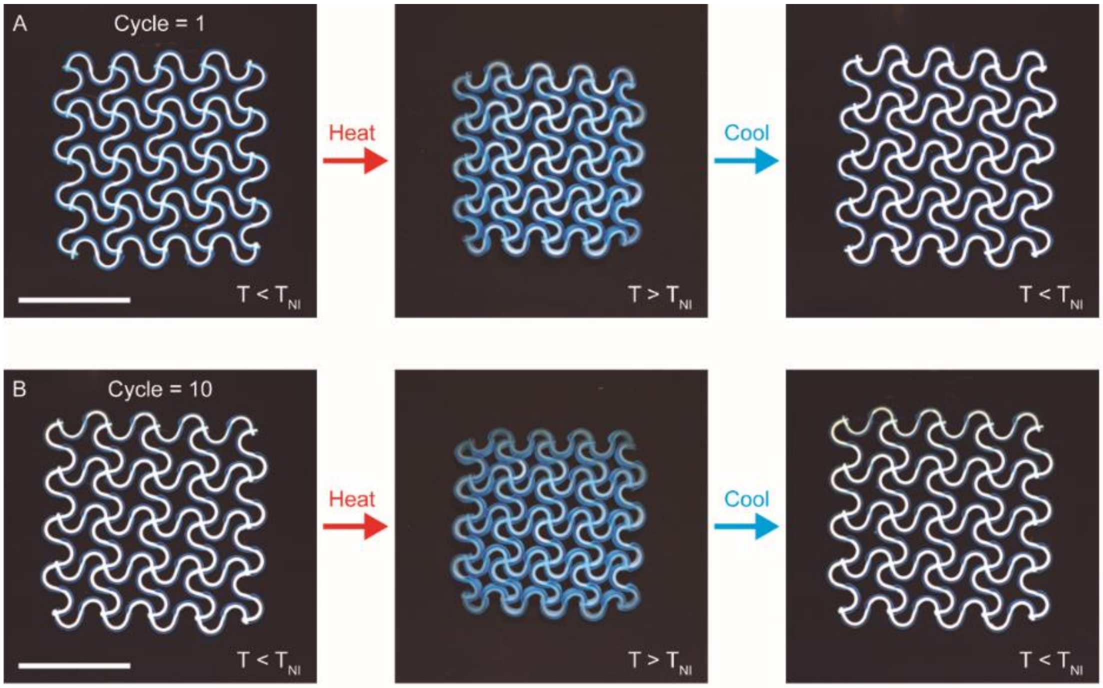

Fig. S16. Cyclic actuation of contracting lattices during the (A) 1st cycle and (B) 10th cycle. Scale bar: 25 mm for both.

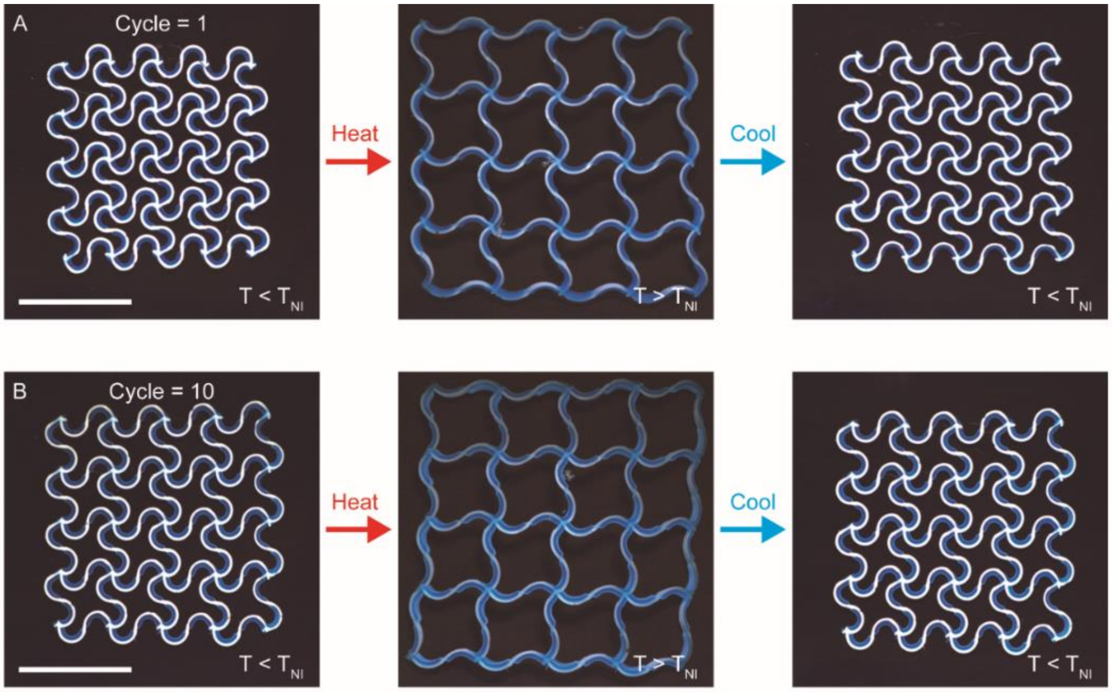

Fig. S17. Cyclic actuation of expanding lattices during the (A) 1st cycle and (B) 10th cycle. Scale bar: 25 mm for both.

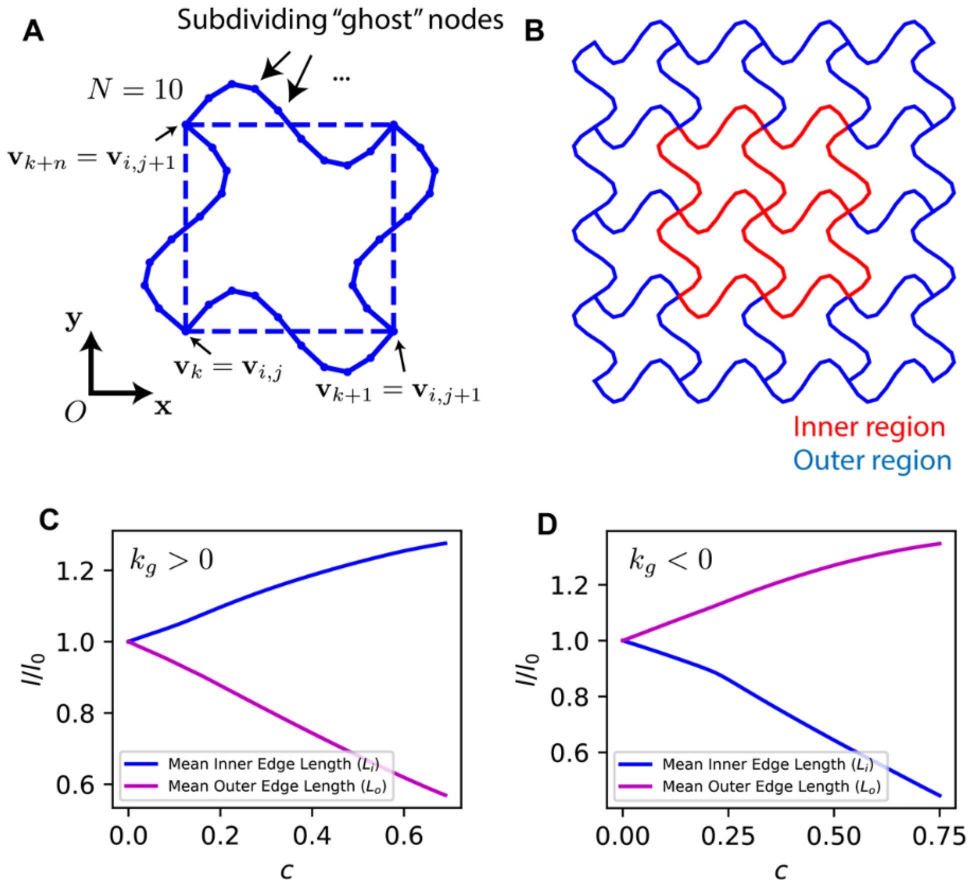

Fig. S18. Discrete parametrization of heterogeneous lattice. (A) Schematic of the meta-graph and the subdivided graph (including ghost nodes) representing the unit cell. Dashed lines indicate the meta-graph unit cell, while solid lines with dots show the subdivided version. (B) Visualization of the inner (red) and outer (blue) unit cells. Evolution of the end-to-end edge length as a function of the curvature change ratio for (C) positively curved and (D) negatively curved lattices.

Movie Captions

Movie S1. Rotational 3D printing of active-passive filaments at a constant print speed of 3 mm s^{-1} while the rotation rate ($\boldsymbol{\omega}^*$) systematically increases from 0 to 0.5.

Movie S2. Active-passive filaments printed at 3 mm s^{-1} with varying rotation rates ($\boldsymbol{\omega}^*$ = 0, 0.06, 0.13, 0.25, 0.5, 1.0, and 2.0) exhibit distinct bending and twisting behaviors upon heating.

Movie S3. Rotational 3D printing of an active-passive lattice at a print speed of 3 mm s^{-1}.

Movie S4. Shape morphing response of an active-passive (expanding) lattice in which the active elastomer positioned on the outer radius of curvature. The lattice expands upon heating and returns to original shape upon cooling.

Movie S5. Shape morphing response of an active-passive (contracting) lattice in which the active elastomer positioned on the inner radius of curvature. The lattice contracts upon heating and returns to the original shape upon cooling.

Movie S6. Shape morphing response of a heterogeneous active-passive lattice (positive gaussian curvature), in which the central unit cells expand, while the outer unit cells contract. When submerged in heated silicone oil, the initially flat lattice morphs into a dome. Simulations confirm the observed deformation.

Movie S7. Shape morphing response of a heterogeneous active-passive lattice (negative gaussian curvature), in which the central unit cells contract, while the outer unit cells expand. Upon heating in silicone oil, the initially flat lattice morphs into a saddle-shaped configuration with negative gaussian curvature, consistent with simulations.

Movie S8. Active-passive lattice filter expands upon heating when submerged in silicone oil allowing 3D objects (e.g., 5-mm spheres or hexagonal nuts) to either be trapped (closed unit cells) or pass through (opened unit cells).

Movie S9. Pick-and-place of multiple objects using an active-passive lattice. A contracting lattice is placed in a silicone oil bath containing an array of 3.5-mm-diameter acrylic rods. Upon heating, the unit cells contract and grip the rods. The rod-filled lattice is then transferred to a new location containing 6-mm-diameter holes. Upon cooling, the unit cells expand and release each rod into its corresponding hole.
